# A Thermoresponsive, Electrically Conductive Bioink Optimized for Electroactive Tissue Engineering and Bioelectronics

**DOI:** 10.1021/acsabm.5c02097

**Published:** 2026-02-15

**Authors:** Róisín Byrne, John Redmond, Keith D. Rochfort, Amanda Carrico, Robert J Forster, Nicholas Dunne, Loanda R Cumba

**Affiliations:** † School of Chemical Sciences, Dublin City University, Glasnevin, Dublin 9 D09 E432, Ireland; ‡ Centre for Medical Engineering Research, School of Mechanical and Manufacturing Engineering, 8818 Dublin City University, Dublin D09 NA55, Ireland; § School of Biotechnology, Dublin City University, Glasnevin, Dublin 9 D09 YT18, Ireland; ∥ Life Sciences Institute, Dublin City University, Dublin 9 D09 YT18, Ireland; ⊥ FutureNeuro, The SFI Research Centre for Chronic and Rare Neurological Diseases, Royal College of Surgeons in Ireland, Dublin 2 D02 YN77, Ireland; # Biodesign Europe, Dublin City University, Dublin 9 D09 YT18, Ireland; ∇ School of Pharmacy, Queen’s University of Belfast, 97 Lisburn Road, Belfast BT9 7BL, U.K.; ○ Advanced Manufacturing Research Ireland Centre (I-Form), School of Mechanical and Manufacturing Engineering, Dublin City University, Dublin 9 D09 YT18, Ireland; ◆ Advanced Materials and Bioengineering Research Ireland Centre (AMBER), Royal College of Surgeons in Ireland and Trinity College Dublin, Dublin 2 D02 YN77, Ireland; ¶ CÚRAM, Research Ireland Centre for Medical Devices, University of Galway, Galway H91 TK33, Ireland

**Keywords:** electrically conductive bioinks, 3D-bioprinting, multicomponent hydrogels, thermoresponsive, biocompatibility

## Abstract

Achieving thermoresponsive behavior, electrical conductivity, printability, and biocompatibility within a single bioink formulation remains a significant challenge, yet this combination is essential for creating stable, electroactive 3D constructs that function under physiologically relevant conditions. To address this unmet need, this study aimed to develop a thermoresponsive and electrically conductive bioink through the systematic formulation and evaluation of 12 hydrogels composed of agarose, gelatin, HPC, and PEDOT:PSS. Among these, a formulation comprising 2% w/w agarose, 4% w/w gelatin, 2% w/w HPC, and 0.1% PEDOT:PSS exhibited the most balanced performance, demonstrating favorable shear-thinning rheology, high print fidelity, structural stability, and high electrical conductivity (0.5757 S/m). Comprehensive biological assays confirmed no significant changes in A549 cell viability across different embedding conditions, while SEM imaging of 3D-printed structures revealed micro- to mesoscale pores suitable for cell infiltration and small molecule transport. Critically, optimizing the PEDOT:PSS content enabled effective conductivity without compromising mechanical properties or biocompatibility. The systematic design approach demonstrated herein provides a reproducible framework for creating multifunctional conductive bioinks that successfully balance thermoresponsive behavior, printability, electrical conductivity, and biocompatibility in a single material. By integration of all essential functional properties into a single formulation, these findings advance the development of application-ready bioinks. The resulting printed structures can be used immediately, without any postprinting modification or functionalization, thereby supporting rapid translation into tissue engineering, biosensing, and bioelectronic applications.

## Graphical abstract

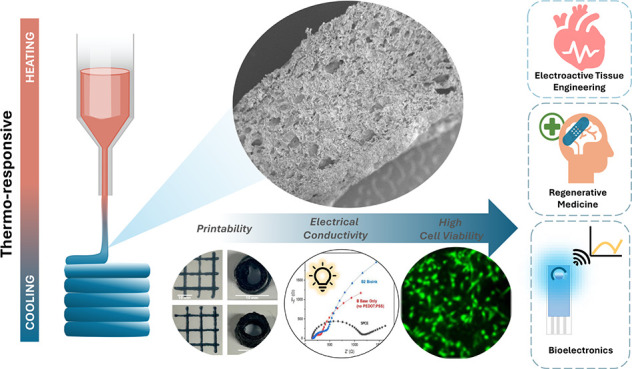

## 1 Introduction

3D bioprinting provides a powerful platform for fabricating complex, spatially organized, tissue-like constructs with precise control over geometry and material composition. Bioinks are typically developed using hydrogel-forming polymers in combination with cells, biomolecules, or functional additives, while aiming to exhibit appropriate printability, structural fidelity, and biocompatibility.^1^ Although many hydrogels can be adapted for use as bioinks, most are intrinsically electrically insulating and therefore are unsuitable for systems that rely on electrical communication or response. Electrically active tissues, such as cardiac and neural models, as well as bioelectronic and biosensing applications, depend on materials that can support ionic and electronic charge transport.^2^ These demands have driven the search for bioinks that integrate biological functionality with electrical performance.

Despite continued progress, many conductive bioinks still rely on high loadings of conductive fillers such as carbon nanotubes, graphene, or polypyrrole, which are prone to aggregation, exhibit poor dispersibility, and can induce cytotoxic effects.^3^ Other approaches enhance conductivity by forming dense polymer networks, but this often impairs cell migration and nutrient transport due to the resulting excessive stiffness.^4^ These trade-offs highlight that printability, mechanical integrity, conductivity, and biocompatibility are interdependent rather than independent design parameters.^5^ In practice, formulations that achieve very high conductivity often do so at the expense of print fidelity or biological integration, which limits their suitability for tissue-relevant or biomedical device applications.^6^ As a result, many conductive bioinks fail to meet the combined requirements for extrusion bioprinting, structural stability, biological functionality, and electrical performance under physiological conditions.^7^

Thermocrosslinkable bioinks offer several advantages for biofabrication, as they form stable networks that maintain mechanical and functional integrity within a biologically relevant temperature range while avoiding harsh cross-linking conditions, such as UV exposure or chemical initiators, that may compromise cell viability.^8,9^ Their thermoresponsive behavior can be tuned by adjusting gel strength (i.e., resistance to deformation under stress), sol–gel transition temperature, polymer concentration, and overall composition.^10^ This tunability is particularly critical as many reported conductive bioinks fail to evaluate or demonstrate stability under physiological conditions. The absence of such validation introduces uncertainty regarding their reliability for cell-laden bioprinting and their subsequent integration within biological environments.^11^ Addressing this issue is essential for applications that require constructs to retain their architecture and performance at around 37 °C.

In this study, we aim to overcome these limitations by developing a multicomponent, thermoresponsive bioink formulation that combines printability, mechanical robustness, electrical functionality, and biological compatibility. Our systematic approach selected polymer components and a cross-linking method that yield a material capable of forming 3D-printed, biomimetic structures with biofunctionality, suitable for advanced regenerative medicine, wearable devices, and bioelectronic interfaces.

The bioinks developed in this study combine two temperature-responsive natural polymers: agarose and gelatin, with the semisynthetic polymer (hydroxypropyl cellulose, HPC), and the conducting polymer PEDOT:PSS (Poly­(3,4-ethylenedioxythiophene)-poly­(styrenesulfonate)). This multicomponent formulation was designed to provide electroconductivity while enhancing mechanical strength, optical transparency, and biodegradability.^12^ Gelatin contains the arginine–glycine–aspartic acid (RGD) motif, which promotes integrin-mediated cellular adhesion, and it gels readily under physiologically relevant conditions.^13,14^ Its mechanical stability is limited, but this can be compensated by combining it with agarose, which can form mechanically robust, thermo-reversible hydrogels with the added potential for self-healing functionality.^15^ Agarose provides an inert and stable environment suitable for encapsulating cells or biomolecules, and it transitions reversibly from a liquid state when heated above its gelation temperature to a stable gel state when cooled, solidifying around 35–40 °C.^16^ Structural robustness and flexibility were further improved by incorporating HPC, which contributes to thermoresponsive gelation and exhibits low cytotoxicity, making it suitable for use in cell-laden formulations.^17^ To introduce electrical functionality, PEDOT:PSS was used due to its high conductivity, chemical stability, and favorable biocompatibility, with PSS acting as a counterion to promote aqueous dispersion and processability.^18,19^ PEDOT:PSS can interact with guanidinium groups on arginine residues in gelatin, enabling electrostatic and π–cation interactions that improve dispersion, homogeneity, and mechanical cohesion within the hydrogel network.^13^

Herein, this study systematically develops and evaluates a series of multicomponent, thermoresponsive bioink formulations by maintaining the HPC content constant while varying the agarose-to-gelatin ratio and the PEDOT:PSS concentration (0.01, 0.1, or 0.5% w/w). The formulations were characterized in terms of swelling behavior, degradation, rheology, and printability to determine their structural fidelity and suitability for extrusion-based bioprinting. The mechanical performance, electrical conductivity, and biocompatibility of the selected formulations were subsequently evaluated. This study establishes a formulation framework for a thermoresponsive, electrically conductive bioink that enables reliable extrusion and stable postprinting performance. By systematically navigating the interplay among mechanical, electrical, and biological constraints, this work advances a formulation space that remains underexplored due to the inherently competing requirements of conductive bioinks. Consequently, it addresses a critical gap in the development of application-ready conductive bioink systems. Among the formulations investigated, several exhibited favorable properties, with one formulation emerging as the most balanced and robust across all evaluated criteria. The optimized material demonstrates the balanced performance necessary for practical use in wearable sensors, soft bioelectronic systems, and physiologically relevant 3D tissue constructs, providing a robust foundation for future integration into more complex bioelectronic or sensing platforms.

## 2 Materials and Methods

### 2.1 Materials

Gelatin type A (from porcine skin, G6144), agarose with low gelling temperature (A9045), and Poly­(3,4-ethylenedioxythiophene)-poly­(styrenesulfonate) (Orgacon, DRY PEDOT:PSS, 768618) were purchased from Sigma-Aldrich (Wicklow, Ireland). Hydroxypropyl cellulose (43400.36) was purchased from Thermo Fisher Scientific (USA). Ultrapure water (molecular biology grade, 693520) used for dissolving the polymers was purchased from Sigma-Aldrich (Wicklow, Ireland). Dulbecco’s modified Eagle’s medium (DMEM, D6429), fetal bovine serum (FBS, F2442), penicillin–streptomycin (P4333), and Trypsin-EDTA (T4049) were purchased from Sigma-Aldrich (Wicklow, Ireland). Phosphate-buffered saline (PBS, P4417) was obtained from Thermo Fisher Scientific (USA). MTT reagent (G3582) from Promega and a lactate dehydrogenase (LDH) assay kit (MAK066) were obtained from Sigma-Aldrich (Wicklow, Ireland). Calcein AM dye (C3099) was purchased from Thermo Fisher Scientific (USA). For degradation and swelling studies, ultrapure water and phosphate-buffered saline tablets (P5517) were purchased from Sigma-Aldrich (Wicklow, Ireland). For the SEM sample preparation, ethanol (EtOH), glutaraldehyde, and hexamethyldisilane were also obtained from Sigma-Aldrich (Wicklow, Ireland).

### 2.2 Multicomponent Electroconductive Bioink Formulation

Initially, PEDOT:PSS was dispersed in ultrapure water at room temperature for 30 min under continuous stirring to ensure complete dissolution. The dispersion process was further aided by sonication using a Fisherbrand Model 120 Sonic Dismembrator: three cycles of one min sonication with one min interval between each cycle, performed at a pulse setting of 05:01 and an amplitude of 50%. The PEDOT:PSS solution was sealed and then gradually heated to 80 °C while being continuously stirred. Agarose was then added, and the mixture was stirred for approximately 20 min to allow complete dissolution. The solution was then cooled to 60 °C, and hydroxypropyl cellulose was added. Upon cooling to 37 °C, gelatin was added and stirred until completely dissolved. In all cases, a homogeneous hydrogel was obtained. Table 1 summarizes the compositions of the electroconductive bioink formulations in which A, B, and C denote different w/w ratios of Agarose:Gelatin.

**Table 1:** Composition of the Bioink Formulations (A1–C3) Contain Different Ratios of Agarose, Gelatin, and Hydroxypropyl Cellulose (HPC), with PEDOT:PSS Varied at 0.01%, 0.10%, and 0.50% (w/w) to Study the Effect of Matrix Composition and Conductive Filler Concentration within Each Series

Bioink ID	%(w/w) PEDOT:PSS	%(w/w) Agarose	%(w/w) Gelatin	%(w/w) HPC
**A1**	**0.01**	**1**	**5**	**2**
**A2**	**0.10**	**1**	**5**	**2**
**A3**	**0.50**	**1**	**5**	**2**
**B1**	**0.01**	**2**	**4**	**2**
**B2**	**0.10**	**2**	**4**	**2**
**B3**	**0.50**	**2**	**4**	**2**
**C1**	**0.01**	**3**	**3**	**2**
**C2**	**0.10**	**3**	**3**	**2**
**C3**	**0.50**	**3**	**3**	**2**

### 2.3 Swelling Analysis

Each ink was heated in a water bath to 45 °C until melted. One mL of each ink was dispensed into a 24-well plate and allowed to solidify at room temperature. The cast hydrogels were then carefully removed and placed in individual Petri dishes. The dry mass was then recorded. The samples were then immersed in 20 cm^3^ of ultrapure water at room temperature over a period of 72 h until equilibrium swelling was reached. The degree of swelling was calculated as a percentage change in weight relative to the weight before exposure to water.

### 2.4 In-Vitro Degradation Tests

Degradation tests were carried out by incubating the hydrogel sample to simulate he physiological conditions. The cast hydrogels were immersed in ultrapure water or 0.01 M PBS in a sealed Petri dish at 37 °C in a temperature-controlled mini-incubator (Labnet) for 10 days. First, the bioink’s initial mass (Wi) was weighed (1.0 g). At each measurement time point, samples were removed from the incubator, and the excess surface liquid was gently removed, weighed, and recorded as (Wt). The degree of degradation was calculated as a percentage change in weight relative to the hydrogel’s initial weight before incubation. Samples were no longer weighed once they had lost their structural integrity. Temperature, solution volume, and storage conditions were monitored daily.

### 2.5 Rheological Characterization

Rheological characterization was performed using an Anton Paar MCR 92 rheometer equipped with a 25 mm diameter parallel plate. Bioink samples (1 mL) were cast into 12 mm Greiner Petri dishes and stored at 4 °C until testing. Prior to analysis, the samples were allowed to equilibrate to room temperature, carefully positioned on the rheometer stage, and trimmed to remove any excess material to ensure a uniform geometry. All rheological experiments were conducted at room temperature unless otherwise stated. A three-interval thixotropy test (3ITT), which mimics the shear conditions experienced during extrusion-based bioprinting, was performed to evaluate the recovery behavior of the bioinks after shear deformation. The test consisted of an initial low shear rate at 1 s^– 1^ for 60 s to establish the baseline viscosity, followed by a high shear rate at 100 s^– 1^ for 5 s to simulate the shear forces encountered during extrusion, and finally a recovery phase using a shear rate of 1 s^–1^ for 120 s to monitor viscosity recovery after shear. Viscoelasticity was determined from oscillatory measurements performed within the linear viscoelastic region using an amplitude sweep in which the shear strain was increased logarithmically from 0.01% to 100% at a constant angular frequency of 10 rad s^–1^, allowing the storage modulus (*G*′) and loss modulus (*G*″) to be recorded to evaluate the elastic and viscous contributions of the bioinks. In addition, a temperature ramp test was carried out to assess thermal stability and flow behavior by heating the samples from 20 to 50 °C at a shear rate of 1 °C every 30 s.

### 2.6 3D Bioprinting Process

The electrically conductive bioink was printed using a CELLINK Inkredible+ bioprinter (CELLINK, Gothenburg, Sweden) equipped with 22G blue standard conical nozzles. The printing was performed under a pressure of 27 kPa and at a temperature of 37 °C. Prior to printing, the bioink cartridges were incubated at 37 °C, and this temperature was maintained throughout the printing process, with the stage held at room temperature. All printed structures were first designed using Fusion 360 software (v2.0.21550, USA). The designs were then exported to Heartware software, which controls the bioprinter.

The printability of the electroconductive bioink was assessed during three distinct constructs as outlined by Schwab et al., by calculating filament spreading ratios, collapse ratios, and pore shape deviations, alongside mean values, standard deviations (SD), and relative standard deviations (RSD) to evaluate consistency and fidelity.^14^ Briefly, filaments were imaged from above, and the filament diameter was measured at three defined intervals (*d1, d2, and d3*) along the central portion of the strand. Mean values, standard deviations, and relative standard deviations (RSD) were calculated to determine reproducibility (Table 2). The RSD, defined as (standard deviation ÷ mean) × 100%, was used as an indicator of variability, with values below 5% generally considered indicative of good printing consistency. Planar structures were analyzed in both top and side views. Filament merging was assessed by printing filaments with a defined spacing, and the actual distances (*d*_1_ and *d*_2_) between the adjacent filaments were measured. The measurements were performed using ImageJ software (NIH, USA).^20^ A multilayer open cylinder structure was printed and analyzed. The adhesion and cohesion between successive printed layers were assessed to evaluate the structural stability of the multilayer architecture.

**Table 2:** Quantitative Print Analysis of Bioink Formulations (A2, B2, C1, and C2)^a^ ^b^ ^c^ ^d^

Formulation	Filament Diameter (mm ± SD) (%RSD)	Grid Spacing (mm)	Pore Area (mm^2^, Avg)	Printing Performance
**A2**	1.675 ± 0.018 (1.08%) 1.340 ± 0.096 (7.15%) 1.527 ± 0.079 (5.18%)	8.13–8.40 (Target 8.5)	69.72 (Target 72.25, 3.5% mismatch)	Slight overextrusion; deformation; partial cylinder collapse
**B2**	1.548 ± 0.038 (2.47%) 1.479 ± 0.058 (3.89%) 1.612 ± 0.063 (3.89%)	8.36–8.42 (Target 8.5)	67.15 (Target 72.25, 7.05% mismatch)	Excellent consistency; slight merging; strong layer stacking
**C1**	1.944± 0.278 (16.65%) 1.530± 0.244 (15.93%) 1.588± 0.255 (16.02%)	7.71–8.31 (Target 8.5)	70.41 (Target 72.25, 2.54% mismatch)	High variability; merging at intersections; lumps at curves
**C2**	1.398 ± 0.013 (0.95%) 1.022 ± 0.023 (2.20%) 1.381 ± 0.114 (8.23%)	8.58–9.35 (Target 8.5)	70.93 (Target 72.25, 1.82% mismatch)	Excellent initial uniformity; later clogging; rougher cylinders

a Filament diameter is reported as the mean ± standard deviation (SD) with the relative standard deviation (%RSD) to evaluate extrusion consistency and reproducibility.

b Grid spacing represents the measured distance between adjacent printed filaments compared to the set target of 8.5 mm, indicating accuracy in spacing and dimensional fidelity.

c Pore area corresponds to the average measured pore size compared with the theoretical target of 72.25 mm^2^, where “% mismatch” compares how close the pore size was to the intended design.

d Printing performance provides a qualitative description of extrusion behavior and overall print fidelity, including filament merging, stacking, uniformity, and structural integrity of the printed constructs.

### 2.7 SEM Analysis

Before SEM imaging, the samples were dehydrated via a graded EtOH series. First, each hydrogel was fixed with 2.5% glutaraldehyde in PBS for 2 h at 4 °C. Gels were then washed twice in dH_2_O for 10 min per wash. Each sample was then passed through an EtOH series consisting of 30, 50, 70, 90, 95, and 100% (x2) EtOH for 10 min per concentration. After the second 100% step, each hydrogel was then transferred to a fresh well plate and submerged in hexamethyldisilazane (HMDS) to improve the drying process. Gels were submerged in 1–2 mL of HMDS for 10 min (x2) before being transferred to a dry Petri dish for overnight air drying. Upon drying, samples were then bisected in various planes and adhered to carbon-coated aluminum SEM stubs. Samples were sputter-coated with gold before SEM imaging was conducted on a Zeiss Mono Cl Evo L515 SEM (Carl Zeiss Microscopy GmbH, Germany) at an accelerating voltage of 5 kV.

### 2.8 Mechanical Testing

Unconfined compression testing was carried out on a CellScale UniVert mechanical tester (CellScale, Waterloo, Canada), fitted with a 100 N load cell. Cylindrical hydrogel samples were prepared by excising discs with a biological tissue hole punch, giving a diameter of ∼10 mm and a thickness of ∼10 mm. Compressive loading was applied at a rate of 15% strain per min, to a maximum strain of 30%, with a preload of 0.01 N. Force and displacement data were recorded. Three sample groups were assessed: dry, wet, and submerged. Dry samples were tested as-prepared under ambient laboratory conditions (∼21.9 °C and 58.5% relative humidity). Wet samples were hydrated in phosphate-buffered saline (PBS) overnight at 4 °C and, prior to testing, lightly blotted to remove excess surface fluid and allowed to equilibrate to room temperature. Submerged samples were tested within a water bath attachment filled with PBS and maintained at 37 °C to simulate physiological conditions. Force and displacement data were continuously recorded and used to generate stress–strain curves, from which the elastic modulus, maximum compressive strength, and toughness (calculated as the area under the curve) were determined.

### 2.9 Electrical Characterization

Electrical conductivity measurements were carried out using a four-point probe Ossila system (Ossila Ltd., United Kingdom). Samples with dimensions of 20 mm × 20 mm × 2 mm were used for the analysis. At room temperature, a constant current was applied between the outer two probes, and the voltage drop was measured across the inner two probes. Sheet resistance, resistivity, and conductivity are reported based on *n* = 100. The probes were gently cleaned with EtOH after each measurement to prevent contamination between the samples.

Electrochemical impedance spectroscopy was performed using a Metrohm Autolab potentiostat/galvanostat equipped with NOVA software (Metrohm Autolab B.V., The Netherlands). Screen-printed carbon electrodes (SPCEs; Metrohm DropSens C11) were used as the working electrode platform. Prior to bioink application, the electrodes were rinsed with deionized water and dried under ambient conditions. The bioink of interest was bioprinted directly onto the working area of the SPCE and allowed to set. EIS measurements were carried out in a solution containing 5 mM K_3_Fe­(CN)_6_ and 100 mM KCl in phosphate buffer (pH 7.4), which was degassed with nitrogen for 15 min prior to measurement to minimize oxygen interference. Impedance spectra were recorded over a frequency range of 0.1 Hz to 100 kHz using a 10 mV AC amplitude at open circuit potential. The data were analyzed using a modified Randles equivalent circuit, including a Warburg element to account for diffusion.

### 2.10 A549 Cell Culture

A549 cells (LGC Standards, UK) were used as a representative cell model for the examination of indices of cell health in response to direct contact with the bioink formulations. Briefly, A549 cells were cultured in Dulbecco’s Modified Eagle’s Medium (DMEM) supplemented with 10% fetal bovine serum, penicillin (100 UmL^–1^) and streptomycin (100 μgmL^–1^). Cells were typically cultured and maintained in 56 cm^2^ Petri dishes with an initial seeding density of 2,000 cells per cm^2^ and maintained in 5% CO_2_/95% humidity at 37 °C. Culture media were replaced with fresh, prewarmed culture media every 72 h until the appropriate confluency was achieved.

### 2.11 Trypsinization of Cells

A549 cultures were typically passaged at approximately 70–90% confluency, as confirmed via light microscopy. All solutions used for subculturing, sterile phosphate-buffered saline (PBS), culture medium, and trypsin/EDTA, were prewarmed to 37 °C in the water bath. In the laminar hood, fresh culture medium was added to new culture dishes and placed in an incubator to equilibrate the medium to 37 °C with 5% CO_2_. For trypsinization, the culture medium on the dishes containing cells was removed using a sterile aspirator. Following this, the culture dish was washed briefly 2–3 times using sterile PBS to ensure the removal of any residual medium. 2 mL of 1x trypsin-EDTA was then added to the culture before the dishes were placed in the incubator for 5–10 min. Cells were routinely observed over this time course until they had displayed a rounded morphology but were not yet fully detached. An equal volume of growth medium to trypsin-EDTA was added to the culture dish, and the cell suspension was then pooled into a 15 mL tube. Following centrifugation at 700 rpm for 5 min, the trypsin-EDTA/culture medium was aspirated, and the resulting cell pellet was gently resuspended via pipetting in 1 mL of fresh culture medium. This cell suspension was then counted using a Neubauer hemocytometer, with trypan blue utilized to determine cell population health. The “viable” count was used when determining volumes of cell suspension for setting up experimental procedures.

### 2.12 Bioink Cell Culture Conditions

Bioinks were stored at 4 °C until use. For assay preparation, precursor solutions were gently warmed to 60 °C and dispensed into sterile 1.5 mL microcentrifuge tubes. The tubes were then UV-sterilized in the UV CLAVE (Benchmark Scientific, USA) for 20 min at 55 °C. After sterilization, samples were transferred to the laminar flow hood and kept at 55 °C until cell seeding. Experiments were performed in 96-well plates at a density of 30,000 cells per well, using four seeding configurations: “Under,” “In,” “In-between,” and “On.”

#### 2.12.1 “Under” Condition

A549 cultures were seeded at a density and allowed to adhere to the dishes for 8 h. The culture media were then removed, and the cells were washed with sterile PBS. 100 μL amount of prewarmed bioink was then added to the well, and the plate was left to incubate at room temperature until polymerization had occurred. 150 μL of prewarmed culture media was then added to the wells, which contained A549 cells, and the plate was returned to the incubator until further required.

#### 2.12.2 “In” Condition

Following cell counting, an appropriate number of cell suspensions were placed into a 1.5 mL microcentrifuge tube, and the cells were pelleted at 700 rpm for 5 min. The culture media supernatant was then removed, and the cells were resuspended in prewarmed bioink at a density of 30,000 cells/100 μL bioink. 100 μL of the bioink/A549 suspension was added to the respective wells, and the plate was left to incubate at room temperature until polymerization had occurred. 150 μL of prewarmed culture media was then added to the wells, and the plate was returned to the incubator for a minimum of 8 h until testing was performed.

#### 2.12.3 “In-Between” Condition

50 μL of prewarmed bioink was added to the respective wells, and the plate was left to incubate at room temperature until polymerization had occurred. A549 cultures were seeded at a density and allowed to adhere to the dishes for 8 h. The culture media were then removed, and the cells were briefly washed with sterile PBS. 50 μL amount of prewarmed bioink was then further added to the respective wells, and the plate was left to incubate at room temperature until polymerization had occurred. 150 μL of prewarmed culture media was then added to the well, which contained A549 cells, and the plate was returned to the incubator until further required.

#### 2.12.4 “On” Condition

A plate was prepared as described above, and A549 cultures were seeded on top of the bioinks and allowed to adhere to the dishes for 8 h. The culture media was then removed, and the cells were briefly washed with sterile PBS. 150 μL of prewarmed culture media was then added to the wells, which contained A549 cells, and the plate was returned to the incubator until further required.

### 2.13 MTS Assays

Following incubation of A549 cultures for the designated amount of time (24–72 h), the MTS assay was used to determine cell viability. 20 μL of a 5 mg/mL solution of 3-(4,5-dimethylthiazol-2-yl)-5-(3-carboxymethoxyphenyl)-2-(4-sulfophenyl)-2H-tetrazolium (MTS) reagent was added to each well, and the plate was then incubated for 4 h at 37 °C in a humidified 5% CO_2_ incubator. 100 μL of the cell culture medium from each well was then transferred to a new 96-well plate, and the absorbance was read at 550 nm on a Tecan Infinite M200 Pro microplate reader. Readings were compared against control wells, which consisted of cells cultured in medium alone without hydrogel, and results were expressed as a percentage relative to these controls.

### 2.14 LDH Assay

Following incubation of A549 cultures for the designated amount of time (24–72 h), the release of stress-related biomarker lactate dehydrogenase was measured to determine potential cytotoxicity. 100 μL of culture media from each well was transferred into the wells of a new 96-well plate. 100 μL of the reaction mixture was then added to each well, and the plate was left to incubate for 30 min at room temperature. Positive and negative controls, whereby A549 cells were treated with 100 nM doxorubicin (positive) and cell culture medium alone (negative), were included. The absorbance was then read at 490 nm on a Tecan Infinite M200 Pro Microplate Reader, and the readings were used to calculate the % cytotoxicity as per the following equation:

1 $$\%\;\mathrm{Cytotoxicity}=\frac{\left(\mathrm{Treated}\;\mathrm{Cell}\;\mathrm{Group}-\mathrm{Healthy}\;\mathrm{Cell}\;\mathrm{Group}\right)}{\left(\mathrm{Doxorubicin‐}\mathrm{Treated}\;\mathrm{Group}-\mathrm{Healthy}\;\mathrm{Cell}\;\mathrm{Group}\right)}\times 100$$

### 2.15 Calcein AM Imaging

Following incubation of A549 cultures for the designated amount of time (24–72 h), calcein AM dye (10 μM) was added to A549 cultures, and the cultures were left to incubate for 4 h. The cultures were then imaged by using a fluorescent microscope to examine cell density and viability in a qualitative manner. Three fluorescence images were taken at random for each condition, and the surface area covered by calcein AM-stained cells was quantified using ImageJ software. The average surface area was calculated and normalized to the corresponding control to determine fold-change values, which are reported as mean ± standard deviation.

### 2.16 Statistical Analysis

All statistical analyses were performed using Origin software (OriginLab Corporation, Northampton, MA). For each formulation, a minimum of three independent replicates (*n* = 3) was tested, with the exact sample sizes reported in the corresponding figure legends. Group comparisons were conducted using one-way or two-way analysis of variance (ANOVA), followed by Tukey’s posthoc test where applicable. Data are expressed as the mean ± standard deviation (SD). Statistical significance was set at *p* ≤ 0.05 (*), p ≤ 0.01 (**), and p ≤ 0.001 (***).

## 3 Results

### 3.1 Swelling and Degradation Assessment

The swelling behavior of all formulations was measured over a 72 h period, and the percentage increase in weight relative to the initial dry weight at 0 h is summarized in Table S1. All formulations exhibited a rapid increase in swelling within the first 24 h, after which most samples reached a plateau or showed only minor changes. The highest degree of swelling was observed in formulation “A2”, which reached 809.6% after 72 h. In contrast, formulation “B3” exhibited the lowest swelling, with a maximum swelling of 399.0% at 72 h. Across the formulations, differences in swelling were evident between classes “A”, “B”, and “C”, with the “A” series generally displaying higher swelling values. The degradation studies were performed in PBS at 37 °C for 10 days following an initial 8 h swelling period. The mass loss profiles for the formulations “A”, “B”, and “C” are shown in Figure 1. Within the “A” series, all formulations exhibited progressive degradation over the 10-day period. In the “B” series, a similar trend was observed, although “B2” retained approximately 50% of its initial mass after 10 days. For the “C” series, samples showed continuous mass loss, with “C3” degrading more rapidly than “C1” and “C2”. Representative images of the hydrogels at Day 0 and after 10 days of incubation are presented in Figure 1, illustrating visible differences in the stability among the formulations.

**Figure 1:**
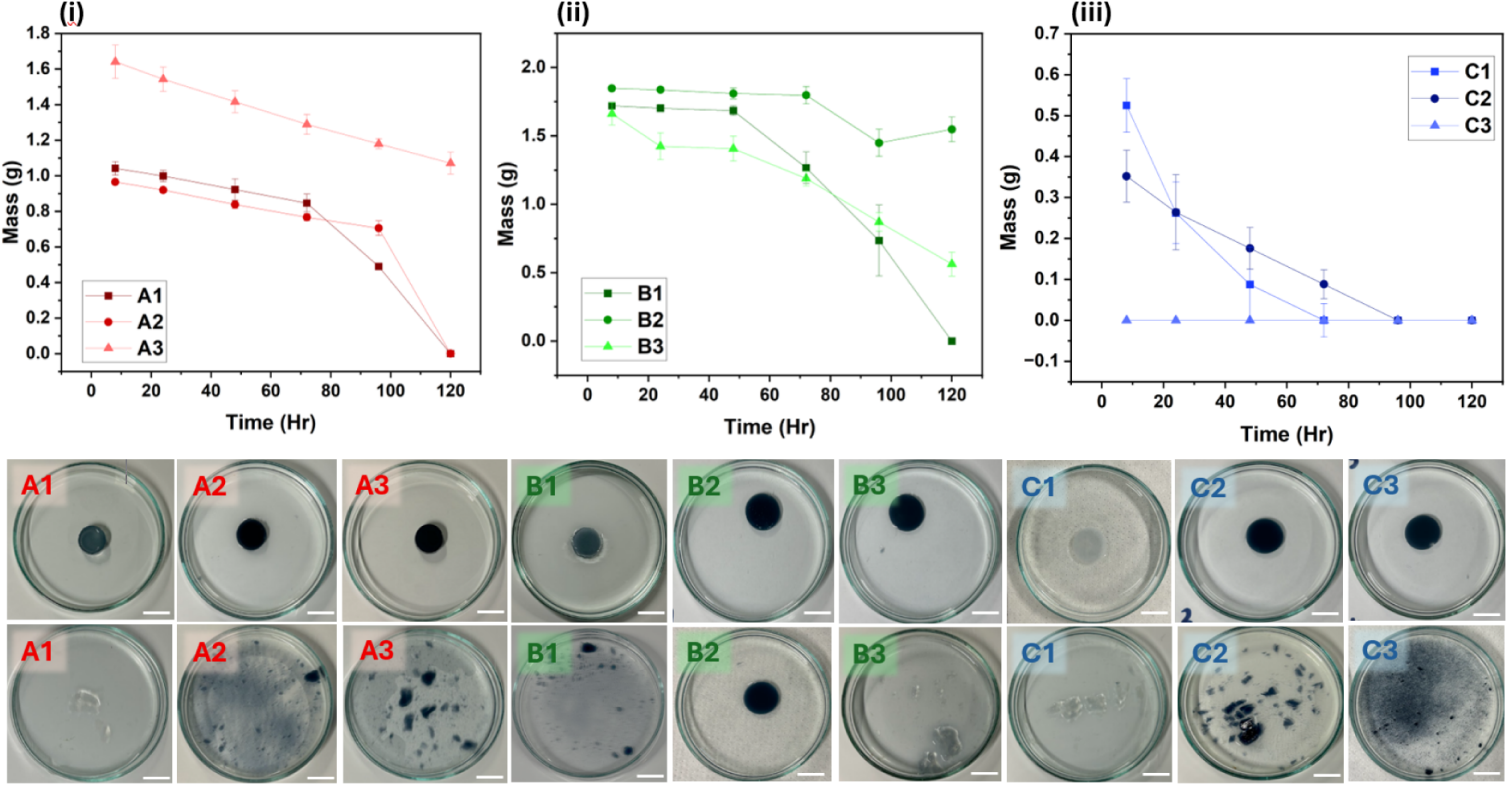
Degradation profiles of (i) formulation “A”, (ii) formulation “B”, and (iii) formulation “C” in PBS at 37 °C over time measured after an initial 8 h swelling period. Data represent the mean ± standard deviation of three independent experiments (*n* = 3). B) Degradation study of bioink formulations “A1”, “A2”,“A3”, “B1”, “B2”, “B3”, “C1”, “C2”, and “C3” in PBS solution at 37 °C. The top row shows the samples at the initial time point (Day 0), while the bottom row shows the same samples after 10 days of incubation. The graphs shown are representative of the run depicted in the photographs below. The scale bar represents 10 mm.

### 3.2 Rheological Evaluation (Thixotropy, Viscoelasticity, Gelation)

The thixotropic response of the formulations was assessed using the three-interval thixotropy test (3ITT), as shown in Figure 2. Across all samples, an initial high viscosity was observed during the first interval, followed by a sharp decrease under shear and partial recovery during the third interval. For the blank formulations (“A”, “B”, and “C”; top row), blank “A” and “B” both displayed similar recovery values of approximately 12.5%, while blank “C” exhibited the highest initial viscosity but the poorest recovery (∼6.7%). For the “A” series (left column), “A2” showed the highest viscosity and the best overall recovery (∼25%), compared to “A1” (∼11.4%) and “A3” (∼12.5%). For the “B” series (middle column), all formulations demonstrated comparable recovery values around 13%, with “B2” exhibiting the highest viscosity within the series. For the “C” series (right column), “C2” displayed the highest initial viscosity but the lowest recovery (∼4.2%), while “C1” and “C3” recovered by approximately 12%.

**Figure 2:**
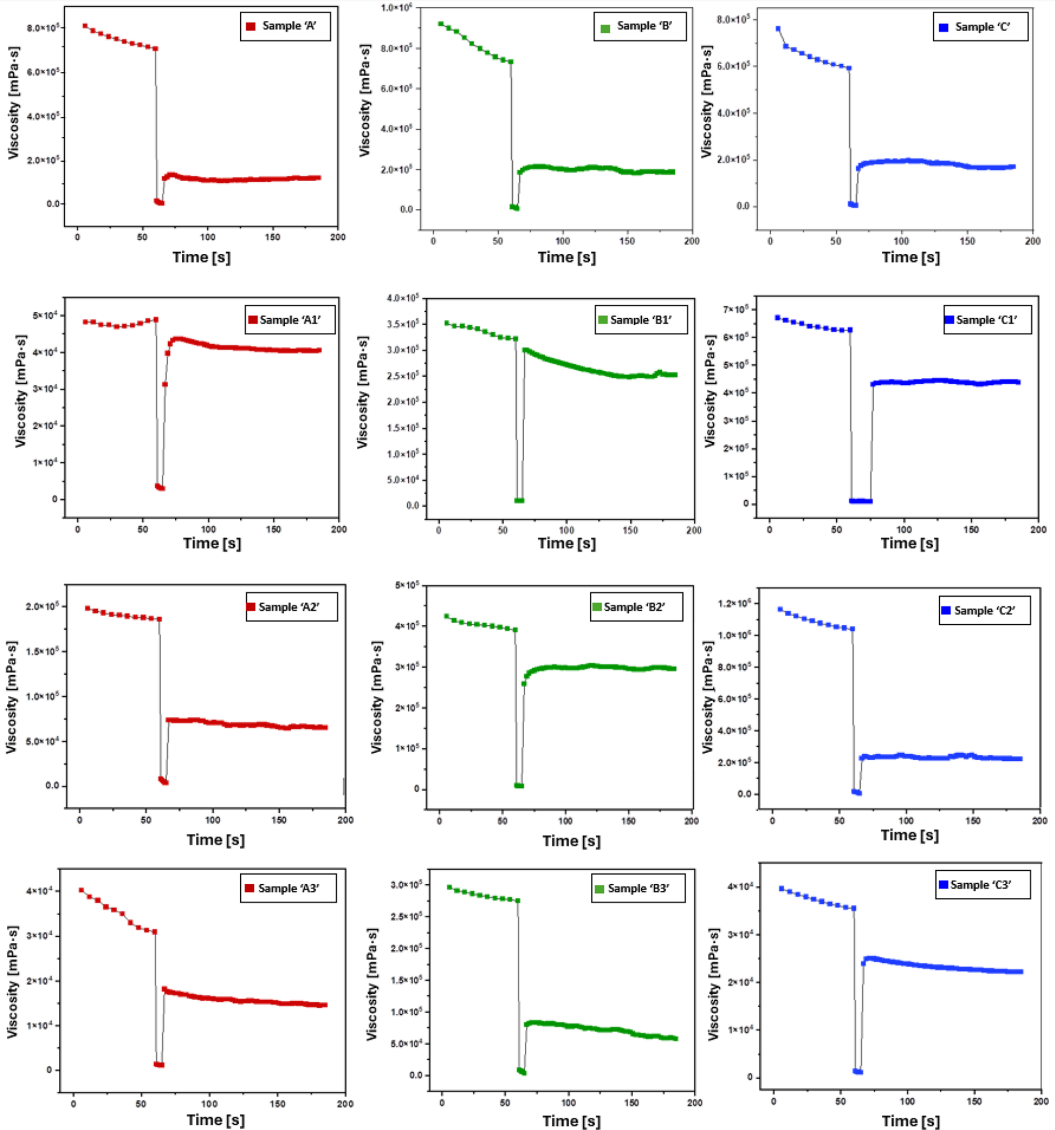
Three interval thixotropic tests of (top row) from left to right: blank sample “A”, blank “B”, and blank “C”. Row 2 denotes samples “A1”, “B1”, and “C1”; row 3 denotes samples “A2”, “B2”, and “C2”; and the final row shows samples “A3”, “B3”, and “C3”, respectively. All measurements were performed on an MCR 92 rheometer and a parallel plate probe. The protocol consisted of an initial shear rate (γ̇) of 1 s^–1^ for 60 s, followed by a higher shear rate interval at 100 s^–1^ for 5 s to mimic extrusion forces, and a final shear rate at 1 s^–1^ for 120 s to assess viscosity recovery after shear.

The viscoelastic behavior of the formulations was assessed by amplitude sweeps, with storage modulus (*G*′) and loss modulus (*G*″) plotted as a function of strain (Figure S2). Across all samples, *G*′ exceeded *G*″ at low strains, confirming predominantly elastic behavior, followed by a decline in modulus values as strain increased. For the blank formulations (“A”, “B”, and “C”; top row), blank “A” exhibited a *G*′ of ∼1.5 × 10^3^ Pa, with crossover occurring near 10% strain. Blank “B” displayed a higher elasticity, with *G*′ of ∼5 × 10^3^ Pa and crossover at ∼30% strain. Blank “C” showed the strongest gel structure, with *G*′ of ∼1 × 10^4^ Pa, maintaining elastic dominance across the widest strain range of the blank samples. For the “A” series (left column), “A1” showed a *G*′ of ∼2 × 10^3^ Pa, with crossover at ∼15% strain. “A2” exhibited a higher *G*′ of ∼6 × 10^3^ Pa, with crossover at ∼25% strain. “A3” displayed reduced elasticity compared to “A1” and “A2”, with *G*′ of ∼1.2 × 10^3^ Pa and crossover occurring below 10% strain. For the “B” series (middle column), “B1” had *G*′ of ∼2 × 10^3^ Pa, with crossover at ∼12% strain. “B2” presented the highest elasticity of the B-series, with *G*′ of ∼8 × 10^3^ Pa and crossover beyond 30% strain. “B3” showed the lowest modulus in the series, with *G*′ of ∼1.5 × 10^3^ Pa and early crossover near 8% strain, marking it as the weakest formulation in terms of viscoelastic stability. For the “C” series (right column), “C1” displayed a *G*′ of ∼7 × 10^3^ Pa, maintaining elasticity until ∼25% strain. “C2” recorded the highest modulus of all tested samples, with *G*′ exceeding 1 × 10^4^ Pa and sustained elastic dominance beyond 40% strain. “C3” showed a lower modulus (∼2 × 10^3^ Pa) with crossover near 10% strain, indicating the weakest viscoelastic stability within the “C” series.

The temperature-dependent viscosity of all formulations was recorded to assess gelation and thermal stability (Figure S3). In all cases, viscosity decreased with increasing temperature, consistent with the thermoreversible behavior of gelatin–agarose systems. For the blank formulations (“A”, “B”, and “C”; top row), blank “A” showed the sharpest transition, with viscosity decreasing rapidly between 26 and 32 °C. Blank “B” exhibited a broader transition across 30–38 °C, while blank “C” displayed the highest initial viscosity and maintained higher viscosity at elevated temperatures compared to blanks “A” and “B”. For the “A” series (left column), “A1” showed a sharp decline between 22 and 28 °C, “A2” transitioned more gradually between 28 and 32 °C, and “A3” exhibited the lowest viscosity and the steepest drop, reaching baseline by ∼30 °C. For the “B” series (middle column), “B1” dropped rapidly between 22 and 30 °C, “B2” demonstrated higher viscosity with a more gradual decline up to ∼40 °C, and “B3” showed the lowest viscosity with a sharper transition than the others. For the “C” series (right column), “C1” decreased steadily between 28 and 40 °C and “C2” maintained the highest viscosity across all formulations and the broadest transition range up to ∼45 °C, while “C3” recorded a lower viscosity and exhibited an earlier transition, beginning near 28 °C.

### 3.3 Printability Assessment

Printability of selected formulations “A2”, “B2”, “C1”, and “C2” was evaluated by comparing printed structures to their corresponding CAD designs (Figure 3). Filament diameters were measured at three points along each filament, and mean values with standard deviations and relative standard deviations (RSD) were calculated to assess reproducibility (Table 2). Formulation “A2” produced filaments with diameters ranging from 1.34 to 1.68 mm, with RSD values of 1.08–7.15%. Printed lines showed deformation and merging, with partial collapse in cylindrical structures, and a pore area mismatch of 3.5% relative to the CAD target. Formulation “B2” yielded diameters of 1.48–1.61 mm, with consistently low RSD values of 2.47–3.89%. Grid spacing (8.36–8.42 mm) closely matched the design target, while pore area deviation was 7.05%. Printed structures showed slight filament merging but strong layer stacking, giving the most consistent printing performance. Formulation “C1” exhibited the highest variability, with filament diameters of 1.53–1.94 mm and RSD values of 15.9–16.7%, leading to uneven deposition, merging at intersections, and irregular surfaces in cylindrical structures. Despite this poor reproducibility, the pore area mismatch was relatively low at 2.54%. Formulation “C2” demonstrated the most uniform filament deposition at the start of printing, with RSD values as low as 0.95%. Variability increased in later prints (up to 8.23%), leading to irregular cylindrical structures; however, the pore area deviation was only 1.82%, making it one of the closest matches to the CAD target.

**Figure 3:**
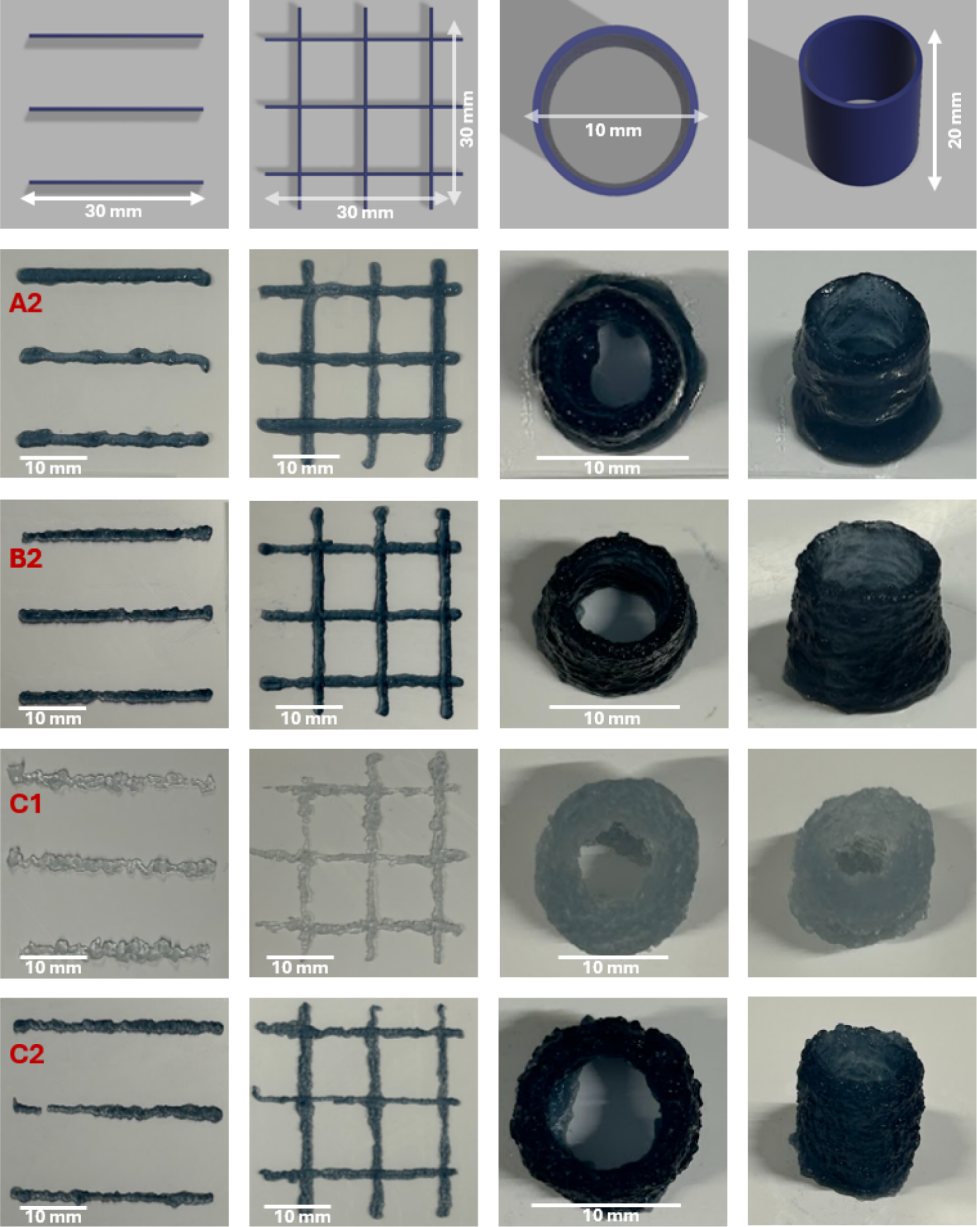
3D-printed structures of bioink formulations (“A2”, “B2”, “C1”, and “C2”) and their corresponding geometries. (Top row) Computer-aided design (CAD) models of the printed line patterns (length: 30 mm, width: 2 mm), grid patterns (30 mm × 30 mm, where the area of the individual squares is *A* = 72.25 mm^2^), and hollow cylindrical structures (10 mm in diameter, 20 mm in height), with dimensions labeled. (Subsequent rows) Images of the printed bioink formulations: (“A2”) 0.1% PEDOT:PSS, 2% hydroxypropyl cellulose, 1% agarose, 5% gelatin; (“B2”) 0.1% PEDOT:PSS, 2% hydroxypropyl cellulose, 2% agarose, 4% gelatin; (“C1”) 0.01% PEDOT:PSS, 3% agarose, 3% gelatin; (“C2”) 0.1% PEDOT:PSS, 2% hydroxypropyl cellulose, 3% agarose, 3% gelatin. The bioinks were printed as single lines, grid patterns, and hollow cylinders (10 mm diameter), demonstrating structural integrity and cross-linking behavior.

### 3.4 Electroconductive Analysis

The electrical properties of the bioink formulations (“A2”, “B2”, “C1”, and “C2”) and blank controls (“A”, “B”, and “C”) were measured using the four-point probe on 20 × 20 × 2 mm hydrogel samples (Table 3). The blank hydrogels were highly resistive, with “A” blank showing the highest sheet resistance (233 kΩ/sq) and lowest conductivity (0.0020 S/m). “B” and “C” blanks recorded lower resistance values (44 and 54 kΩ/sq, respectively), with correspondingly higher conductivities up to 0.0087 S/m. Incorporation of PEDOT:PSS markedly improved electrical performance across all formulations. Among these, “B2” exhibited the lowest sheet resistance (0.68 kΩ/sq), lowest resistivity (1.74 Ω·m), and highest conductivity (0.58 S/m). “A2” also showed an improvement over its blank, with a conductivity of 0.063 S/m. The “C” series formulations displayed intermediate values, with “C1” reaching 0.062 S/m and “C2” increasing further to 0.23 S/m.

**Table 3:** Electrical Properties of Bioink Formulations and Blank Controls Measured via a Four-Point Probe^a^

Four-point probe			
Sample	Sheet Resistance (kΩ/sq)	Resistivity (Ω·m)	Conductivity (S/m)
**A Blank**	233.00 ± 34.94 (3.9%)	494.01 ± 19.39 (3.9%)	0.0020 ± 0.00 (3.9%)
**B Blank**	44.40 ± 1.40 (3.3%)	94.10 ± 3.15 (3.3%)	0.0106 ± 0.00 (3.4%)
**C Blank**	54.40 ± 8.16 (1.5%)	115.00 ± 1.7 (1.5%)	0.0087 ± 0.00 (1.5%)
**A2**	7.93 ± 0.05 (0.7%)	15.90 ± 0.00 (0.7%)	0.0630 ± 0.00 (0.7%)
**B2**	0.68 ± 0.02 (3.2%)	1.74 ± 0.058 (3.2%)	0.5757 ± 0.03 (4.5%)
**C1**	16.30 ± 2.44 (6.0%)	16.30 ± 0.98 (6.0%)	0.0615 ± 0.00 (6.0%)
**C2**	3.11 ± 1.03 (3.3%)	4.36 ± 0.14 (3.3%)	0.230 ± 0.07 (3.3%)

a Sheet resistance (KΩ/square), resistivity (Ω·m), and conductivity (S/m) and the mean ± standard deviation (SD) with relative standard deviation (%RSD) are shown.

### 3.5 Mechanical Assessment

The compressive properties of formulation “B2” were evaluated under three conditions: dry, wetted, and submerged (Figure 4). The ultimate compressive strength (UCS) increased with hydration, from 12.68 ± 1.75 kPa in the dry state to 15.16 ± 1.08 kPa when wetted, and 17.09 ± 0.81 kPa under submerged conditions. The elastic modulus also rose significantly with hydration, with values of 87.11 ± 19.59 kPa (dry), 113.29 ± 10.03 kPa (wetted), and 119.28 ± 9.80 kPa (submerged). Statistical analysis indicated that the increase from dry to wetted and dry to submerged was significant (*p* < 0.05), while no significant difference was observed between the wetted and submerged conditions (*p* = 0.32). Toughness values followed a similar trend, increasing from 0.0187 ± 0.0048 J/m^3^ (dry) to 0.0233 ± 0.0049 J/m^3^ (wetted) and 0.0270 ± 0.0009 J/m^3^ (submerged). A statistically significant difference was observed between the dry and submerged states (*p* = 0.007), while the change between dry and wetted states was not significant.

**Figure 4:**
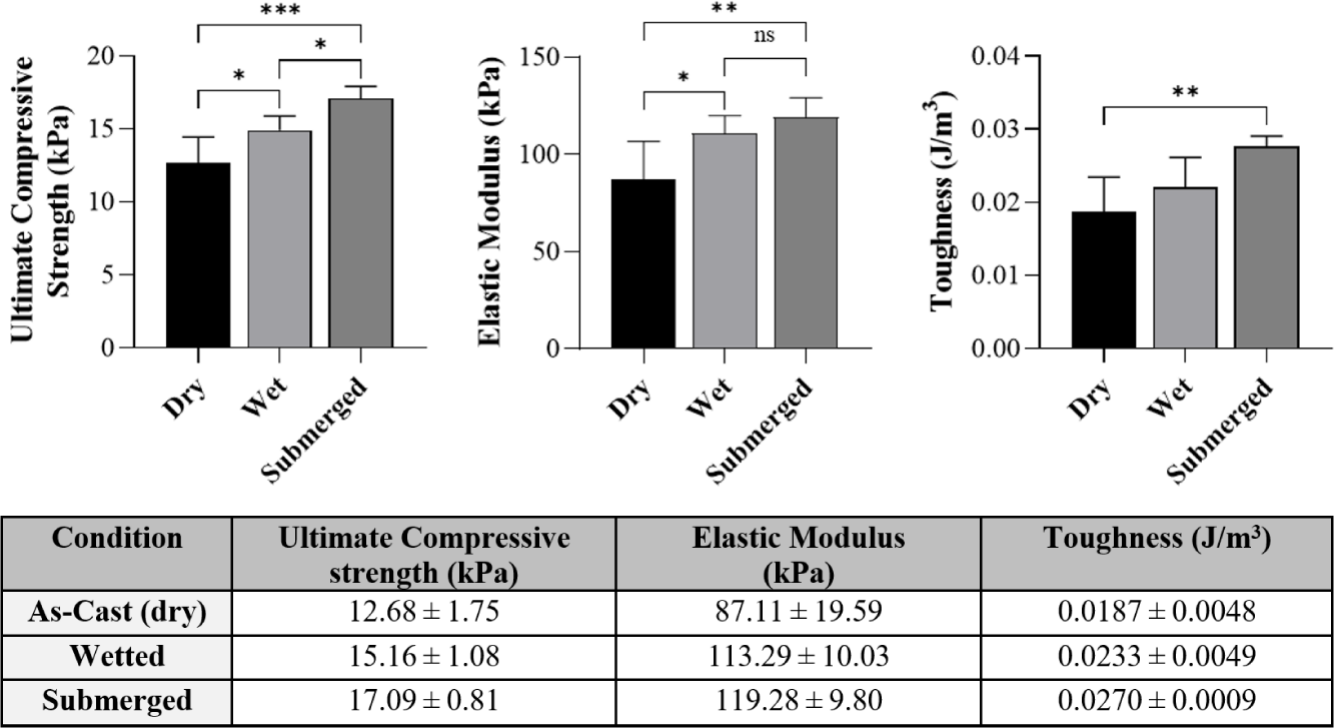
Mechanical properties and tabulated averages of the “B2” bioink under different hydration states. Graphs (top) display the ultimate compressive strength, elastic modulus, and toughness of the bioink in as-cast (dry), wetted, and submerged conditions. Statistical significance is denoted as *p* < 0.05 (*), *p* < 0.01 (**), and *p* < 0.001 (***), while “ns” indicates no significant difference. The table (bottom) summarizes the corresponding mean ± standard deviation values for each property across conditions (*n* = 3).

### 3.6 Scanning Electron Microscopy (SEM)

SEM imaging was performed on the dehydrated “B2” bioink formulation (Figure 5). At low magnification (Figure 5D), the cross-section showed a porous architecture distributed throughout the hydrogel. At 100 μm magnification (Figure 5C), an interconnected network of pores was observed, with pore sizes ranging from approximately 40–100 μm. At higher magnifications (50 and 40 μm, Figure 5B and 5A), the surface morphology appeared rough and heterogeneous, with visible irregularities and variations in texture. The pore walls displayed uneven features, and regions of varying density were evident across the structure.

**Figure 5:**
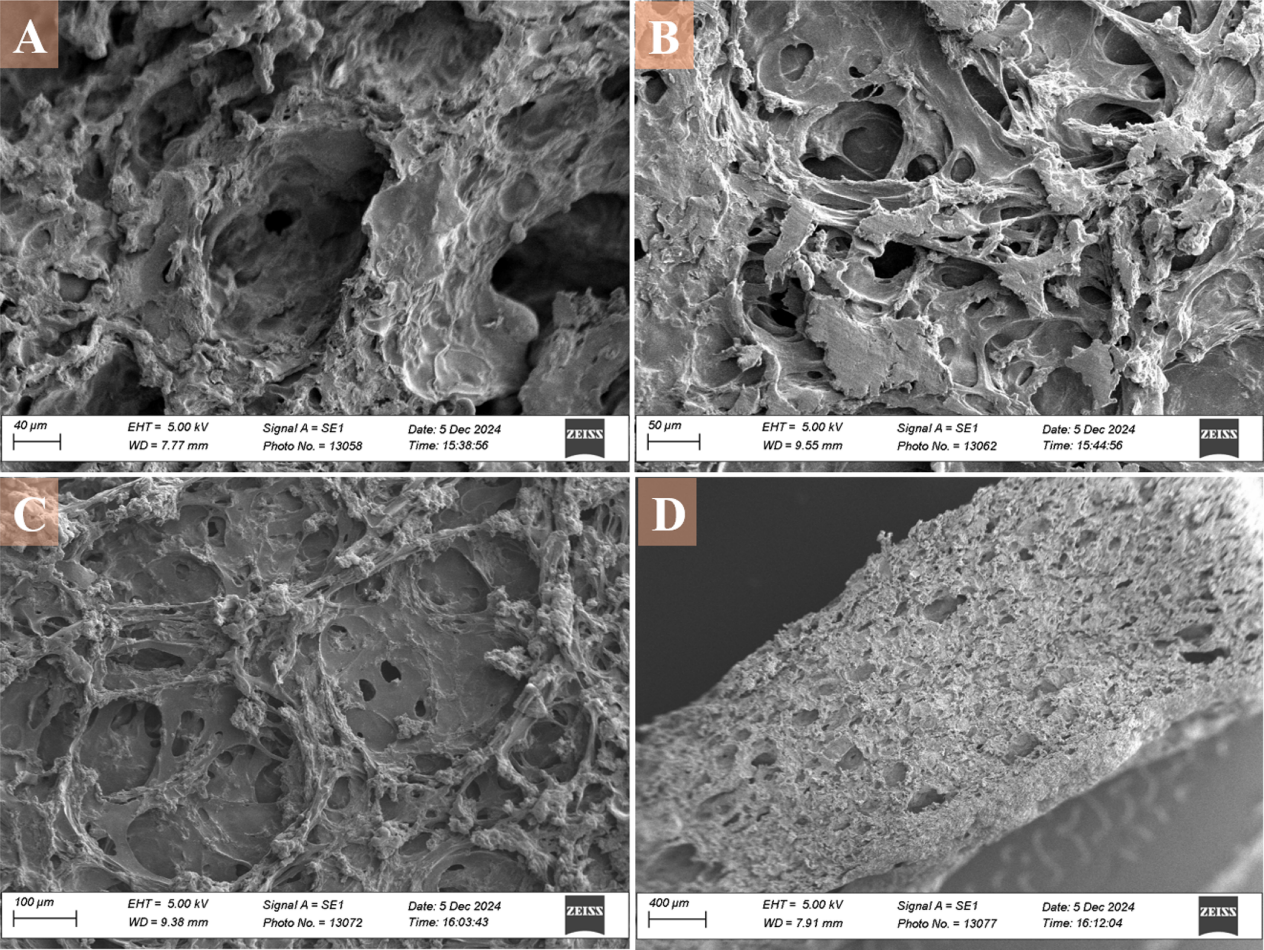
SEM images of the “B2” bioink formulation (4% gelatin, 2% agarose, 2% hydroxypropyl cellulose, and 0.1% PEDOT:PSS) after ethanol dehydration at various magnifications. A: Surface morphology at the 40 μm scale. B: Porous structure at a 50 μm scale. C: Network structure highlights interconnected pores at a 100 μm scale. D: Cross-sectional view at a 400 μm scale, showing the internal architecture and porosity of the bioink.

### 3.7 Electrochemical Impedance Spectroscopy (EIS)

Electrochemical impedance spectroscopy was used to evaluate the interfacial electron transfer behavior of the “B2” bioink, the base hydrogel without PEDOT:PSS, and the bare screen-printed carbon electrode (SPCE) (Figure 6). In the Nyquist plot (Figure 6, left), the unmodified SPCE (black squares) displayed the largest semicircle, corresponding to the highest charge transfer resistance (*R*_ct_ ∼ 1.2 kΩ). The base hydrogel without PEDOT:PSS (red circles) exhibited the smallest arc, indicating the lowest *R*_ct_ (∼220 Ω) among the tested samples. The “B2” bioink (blue triangles) produced an intermediate response, with a charge transfer resistance of ∼522 Ω. In addition, the low-frequency Warburg region of the “B2” bioink showed a more extended slope compared to the base hydrogel, reflecting greater diffusional impedance. The Bode plot (Figure 6, right) further highlighted the differences in frequency-dependent impedance. Across the frequency range, the SPCE showed the highest overall impedance values, while the base hydrogel exhibited the lowest. The “B2” bioink consistently displayed intermediate values, with impedance decreasing steadily at higher frequencies.

**Figure 6:**
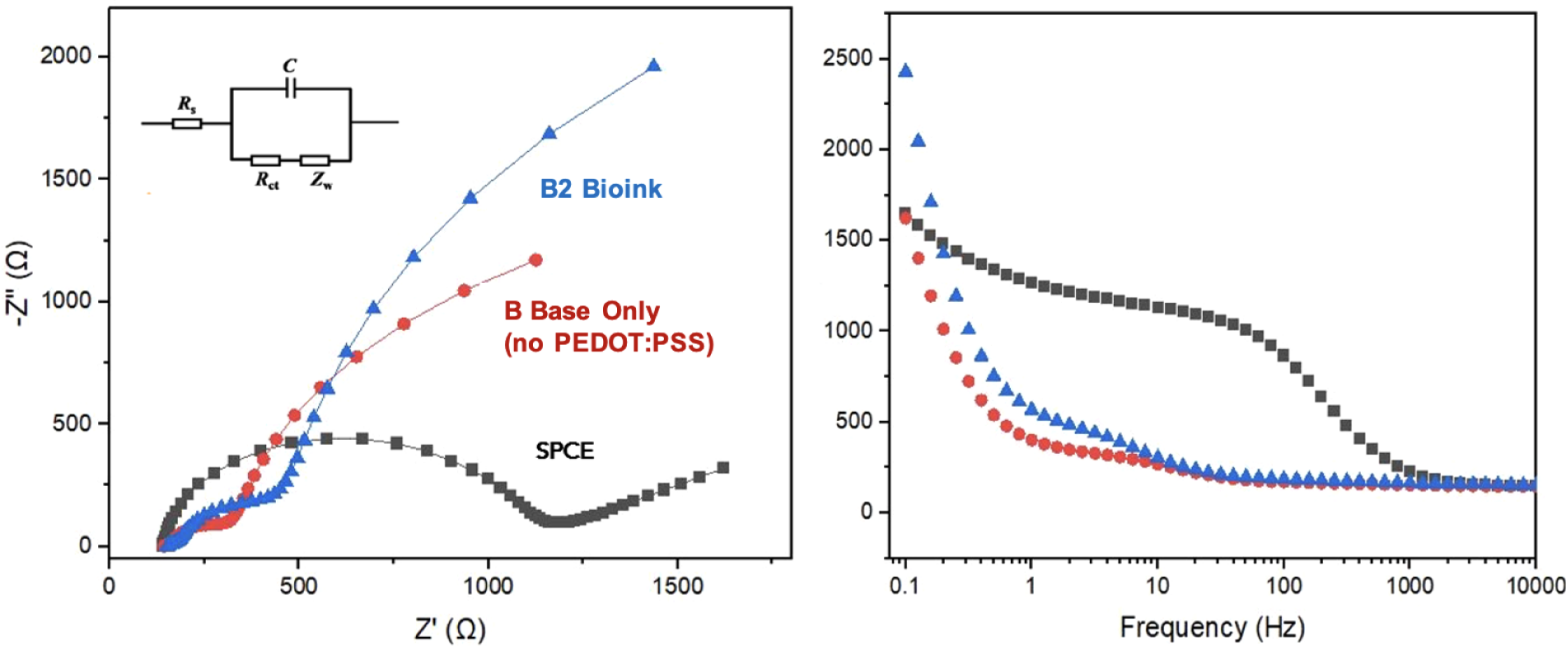
Electrochemical Impedance Spectroscopy (EIS) analysis comparing the “B2” bioink (blue triangles), “B” base formulation without PEDOT:PSS (red circles), and unmodified screen-printed carbon electrode (SPCE, black squares). Measurements were conducted in a solution of 5 mM K_3_Fe­(CN)_6_ and 100 mM KCl prepared in phosphate buffer (pH 7.4). The Bode plot (right) highlights the frequency-dependent impedance behavior across formulations.

### 3.8 Biocompatibility Evaluation

Cell viability and any potential cytotoxicity were assessed under four experimental conditions: cells seeded directly on top of the hydrogel surface (“On”), beneath the hydrogel (“Under”), between two hydrogel layers (“In-between”), and incorporated within the hydrogel during gelation (“In”) (Figure S1). Viability was measured by the MTS assay (Figure 7i–iv), and cytotoxicity was assessed by the LDH assay (Figure 7v–viii). Across all conditions, untreated hydrogels (Control, “B”, “B2”) maintained high cell viability over the 72 h testing period. One-way ANOVA showed no significant effect of time for Control and “B” (*p* > 0.05). “B2” was the only sample to show an overall time effect (*p* < 0.05), but posthoc analysis indicated no significant pairwise differences between time points, suggesting variability was minor. Comparisons between materials at each time point showed no significant differences between non-DOX groups (Control vs “B”, Control vs “B2”, “B” vs “B2”; *p* > 0.05). In contrast, all DOX-containing groups (Control/DOX, “B”/DOX, “B2”/DOX) showed significantly reduced viability compared with their non-DOX counterparts at each time point (*p* < 0.01), consistent across all four conditions. The LDH assay revealed similar trends. Untreated hydrogels showed low cytotoxicity values with no significant effect of time (*p* > 0.05). “B2” again displayed an overall time effect (*p* < 0.05), but posthoc comparisons were not significant, indicating no distinct time point differences. Comparisons between materials confirmed no significant differences between untreated hydrogels (*p* > 0.05). In contrast, all DOX-containing samples exhibited significantly higher cytotoxicity compared to non-DOX groups at every time point (*p* < 0.001).

**Figure 7:**
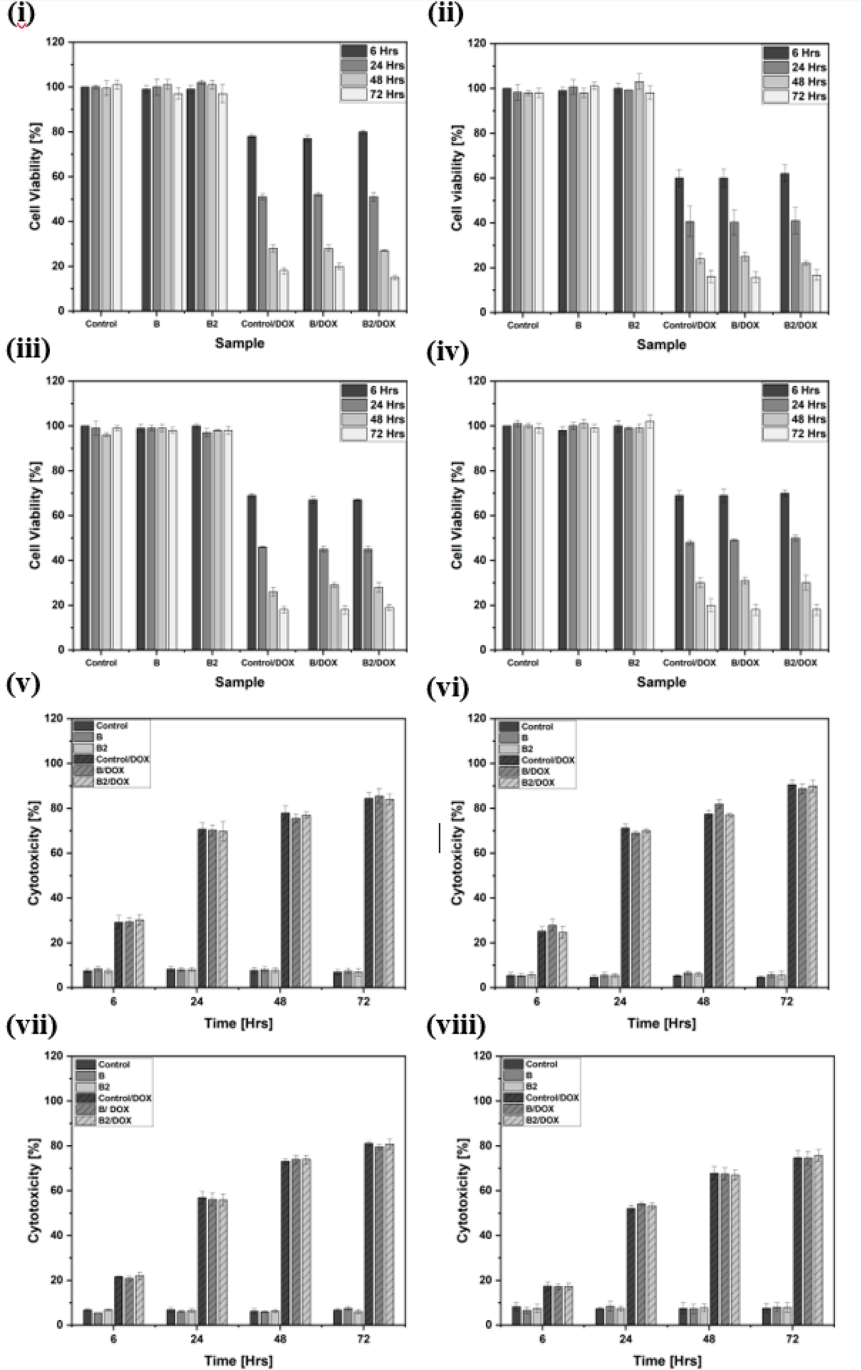
MTS and LDH assay results for cell viability and cytotoxicity under varying sample conditions: (i, v) “on” condition, where cells were deposited on top of the hydrogel sample; (ii, vi) “under” condition, where cells were deposited beneath the hydrogel sample; (iii, vii) “in-between” condition, where cells were deposited on half of the hydrogel sample and pressed with the other half; and (iv, viii) “inside” condition, where cells were mixed homogeneously within the hydrogel. For each condition, the top row (i–iv) shows cell viability (MTS assay), and the bottom row (v–viii) shows cytotoxicity (LDH assay). In each graph, bars represent either material-dependent results (Control, “B”, “B2”, and their DOX-treated variants) or time-dependent results (6, 24, 48, and 72 h). Cell viability and cytotoxicity are expressed as percentages where results are averaged from three independent experiments, *n* = 3 ± SD.

Fluorescence microscopy images (Figure 8) using Calcein-AM live cell staining supported these findings. Strong green fluorescence was observed for Control, B, and B2 across all conditions with no visible differences between groups, indicating high viability. In DOX-containing groups, fluorescence intensity was markedly reduced across all conditions with no observable difference between DOX-treated materials, confirming reduced cell viability consistent with the cytotoxic action of doxorubicin. Quantitative fold-change analysis of fluorescence intensity further supports these observations and is summarized in Table S2.

**Figure 8:**
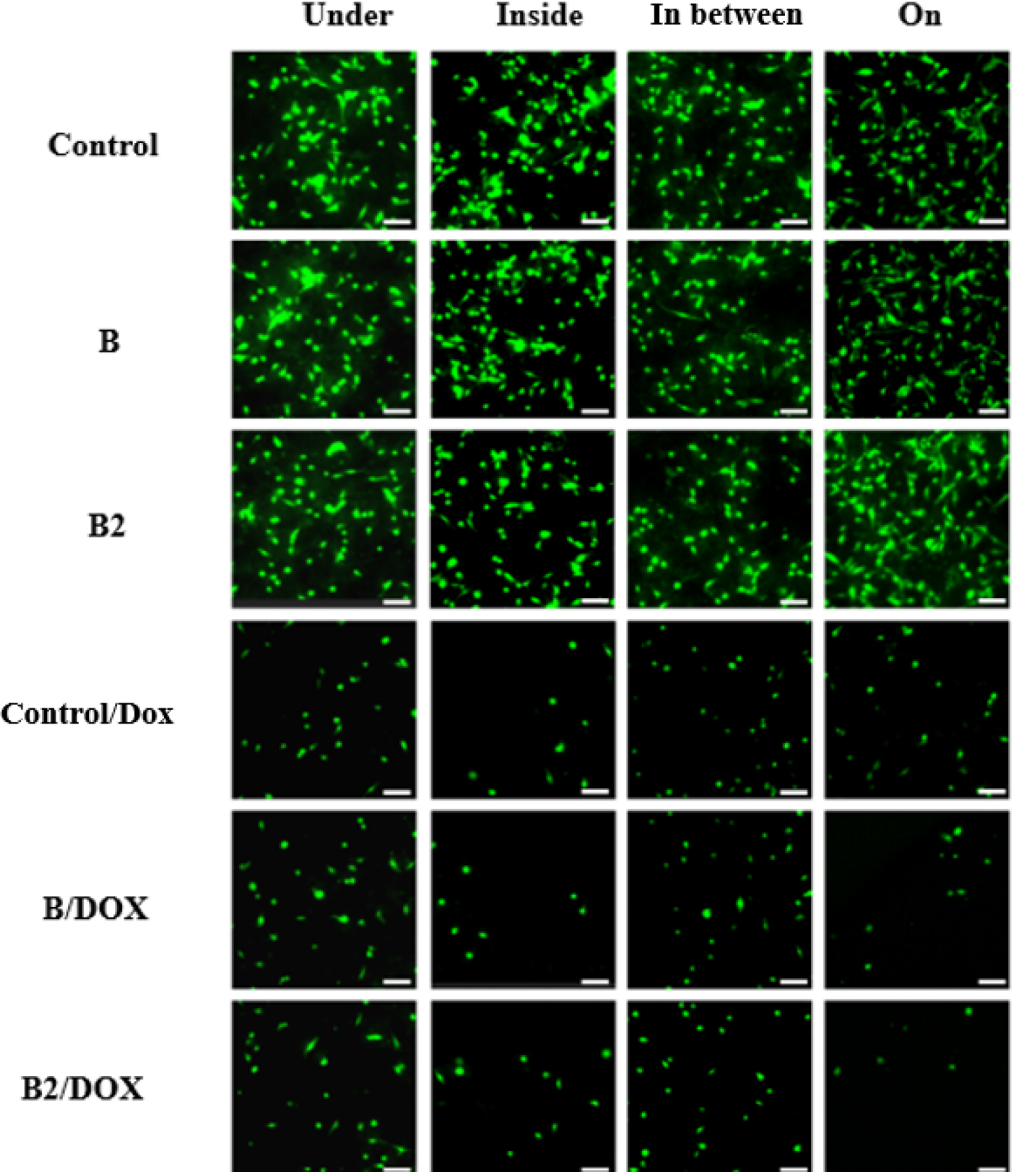
Fluorescence microscopy images of live cell staining using Calcein-AM, showing qualitative cell viability under different hydrogel conditions. The columns represent the four experimental setups: “Under,” “Inside,” “In-between,” and “On.” The rows correspond to different material compositions: Control, “B”, “B2”, and their respective DOX-treated variants (Control/DOX, B/DOX, B2/DOX). Live cells fluoresce green, indicating metabolically active cells. These images are intended as qualitative visual support for the viability trends observed in the quantitative assays; no cell counting was performed. The scale bar represents 50 μm.

## 4 Discussion

3D bioprinting has emerged as a powerful platform for advancing tissue engineering, biosensing, and drug delivery applications.^1^ Despite significant progress, the field remains constrained by the limited availability of bioinks that are not only biocompatible and structurally stable but also electrically conductive and multifunctional. Developing such bioinks is challenging, as the introduction of conductive fillers can improve charge transport but often alters viscosity, printability, and cell compatibility in a concentration-dependent manner.^5^ Cross-linking adds further complexity, as extrusion-based inks must balance low viscosity for deposition with sufficient postprinting stability and cell bioactivity. Harsh chemical, enzymatic, or photo-cross-linking methods can compromise sensitive biological components, whereas physical cross-linking via reversible noncovalent interactions offers milder, adaptable conditions.^21^ Thermoresponsive hydrogels are particularly attractive, as small temperature shifts near physiological conditions enable sol–gel transitions that support gentle extrusion and rapid postprinting solidification.^10^ This mild, reversible gelation is particularly advantageous for bioinks containing delicate components such as proteins, antibodies, or living cells as it preserves their functionality while maintaining a supportive environment for growth and bioactivity. For example, Kim et al. developed a thermoresponsive bioink designed for personalized scaffolds that enable antioxidant release and fibroblast delivery to accelerate diabetic wound healing. This system allowed gentle cell encapsulation and preserved cell viability during the printing process, demonstrating strong structural and biological functionality.^22^ However, its design was limited in scope as it did not incorporate electrical conductivity, an important factor that could further enhance tissue regeneration and wound healing outcomes. Similarly, Garcia-Hernando et al. fabricated an electroactive and thermoresponsive material for the capture and release of cells that demonstrated the potential of combining charge transport with reversible gelation, but it was not optimized as a printable bioink.^23^ Together, these examples illustrate that while significant progress has been made toward thermoresponsive or electrically conductive materials, there remains a gap in integrating these properties into a single, printable, biocompatible bioink. The present study addresses this by producing and outlining a design strategy for electrically conductive, thermoresponsive hydrogels that balance printability, conductivity, and biological compatibility.

Swelling and degradation behavior are critical indicators of hydrogel performance, as they determine both nutrient/analyte diffusion and long-term structural stability.^24^ For this reason, these parameters were evaluated as an initial step in assessing the multifunctionality of the developed bioinks since their ability to withstand such conditions provides an early indication of their suitability under physiologically relevant environments. The swelling behavior of the hydrogels reflected the influence of both polymer composition and PEDOT:PSS concentration, as summarized in Table S1. Swelling was most pronounced in formulation “A2”, which contained the highest gelatin concentration (5% w/w). In contrast, samples with elevated PEDOT:PSS levels (0.5% w/w) showed markedly reduced swelling likely due to stronger cross-linking interactions between PEDOT:PSS and gelatin that limited water uptake and network expansion. This behavior is consistent with reports by Furlani et al., who observed enhanced cross-linking density and reduced swelling with increasing PEDOT:PSS content in gelatin-PEDOT:PSS-based systems.^13^ Degradation analysis further highlighted the influence of filler concentration and polymer ratios, with all samples exhibiting varying degrees of mass loss but showing a consistently gradual decline over time, indicating ongoing degradation, as seen in Figure 1. Higher agarose content demonstrated greater long-term stability, while those rich in gelatin degraded more rapidly under hydrolytic conditions. Among the PEDOT:PSS-containing formulations, “B2” (0.1% w/w PEDOT:PSS) was the most stable, retaining approximately half of its mass after 10 days while maintaining its structural integrity, visually represented in Figure 1. The improved stability at this intermediate concentration may arise from the balanced contributions of agarose rigidity, gelatin-mediated water uptake, and PEDOT:PSS reinforcement. By contrast, higher PEDOT:PSS loading accelerated degradation, consistent with the increased brittleness reported in other conductive hydrogels.^25^ Notably, sample “C3” could not withstand the initial swelling phase and had completely fragmented before 72 h, preventing further accurate mass measurements; this is reflected by the flat line in the graph, indicating no measurable change thereafter. Furthermore, similar concentration-dependent effects were described by Spencer et al. in GelMA–PEDOT:PSS composites, where mass loss increased with rising PEDOT:PSS content (<0.3%).^26^

Thixotropy, viscoelasticity, and gelation are critical features in bioink design since inks must withstand shear forces during extrusion yet rapidly recover structural integrity after deposition, all under conditions compatible with cell viability.^27^ Recovery capacity varied with polymer composition, as observed in Figure 2, where samples containing high agarose content showed strong initial viscosities but limited network regeneration, while formulations containing more gelatin recovered more effectively but were prone to lower mechanical stability. Dravid et al. examined the rheology of different agarose-gelatin blends and reported that agarose-rich inks demonstrated higher initial viscosities and stronger elastic networks, whereas gelatin-rich inks were more dynamic but mechanically weaker, mirroring the above findings.^28^ However, the study was conducted by Dravid et al., is limited to electrically insulating materials, and therefore does not address the impact of conductive fillers on rheological behavior. The present work extends on these observations by demonstrating comparable rheological and biocompatibility profiles, while introducing an electrically conductive component such as PEDOT:PSS. At low concentrations (0.01% and 0.1% w/w), PEDOT:PSS enhanced recovery, most likely through reversible electrostatic and π–cation interactions between PEDOT and gelatin residues that promote network cohesion.^26^ This is clearly evident in the direct comparison between the control sample “A” and sample “A1” as seen in the first two graphs in the first column of Figure 1, where even with only 0.01% w/w PEDOT:PSS, sample“A1” exhibited a noticeably higher recovery profile. By contrast, high PEDOT:PSS loading (0.5%) consistently disrupted regeneration, indicating that excess polymer interfered with uniform network formation and promoted brittleness, a behavior also reported in other conductive hydrogels.^29^ The viscoelasticity tests in Figure S2 further corroborated the thixotropy analysis. The magnitude and stability of *G*′ within the linear viscoelastic region gradually improve moving from row one (blank samples “A”, “B”, “C”) to rows two and three (“A1”/”B1”/“C1” and “A2”/“B2”/“C2”), suggesting enhanced network strength. For example, the agarose-rich formulation “C2” exhibited the highest initial viscosity (∼1.2 × 10^6^ mPa·s) and the strongest gel structure (*G*′ > 1 × 10^4^ Pa) but recovered poorly (∼4% of its original viscosity), reflecting the rigidity imparted by agarose. By contrast, formulation “A2” displayed the greatest recovery (∼25%), while maintaining a more gradual decline in both *G*′ and *G*″ beyond the critical strain point, suggesting that a composition enriched in gelatin provided the most favorable balance between structural stability and network reformation. Temperature gelation sweeps as seen in Figure S3 confirmed the thermoresponsive nature of the blends, with gelatin-rich samples showing sharp sol–gel transitions near physiological temperature (e.g., “A2”), while agarose-rich samples transitioned more gradually and maintained viscosity over broader ranges (e.g., “C3”), as described previously for agarose/gelatin systems by Dravid et al.^28^ Formulations containing 0.5% w/w PEDOT:PSS such as “C3” also seemed to produce a smoother sol–gel transition when compared to lower concentrations. The additional PEDOT:PSS likely reduces the sharpness of the gelation point by interfering with polymer–polymer interactions, effectively broadening the transition window.^30^ Given that both rheological performance and thermoresponsiveness can significantly influence cell survival in bioink matrices, it is valuable to compare the present findings with other temperature-sensitive hydrogel systems. Gu et al. developed a thermoresponsive agarose-based bioink that exploited a cooling step during printing to stabilize constructs.^31^ The agarose-based bioink demonstrated high-density cell encapsulation (3 × 10^7^ cells mL^–1^) with postprinting viabilities exceeding 90%, providing strong evidence that such thermoresponsive transitions are compatible with biological function. This study highlighted strong parallels with the present work, demonstrating that thermoresponsive bioinks can accommodate temperature shifts during processing without loss of biocompatibility, thereby reinforcing their suitability for extrusion-based bioprinting.

The results from the swelling and degradation studies readily identified formulations that were unsuitable for further development, and these were subsequently refined by rheological assessment to determine those most appropriate for advanced characterization. Based on this process, the formulations carried forward included “A2”, “B2”, “C1”, and “C2”. Depicted in Figure 3, these selected samples were then evaluated for their printability, a critical criterion in determining their suitability for extrusion-based bioprinting.^14^ Overall, “B2” offered the most reliable performance, with low RSD values (2.47–3.89%), grid spacing closely matching the CAD design, and printed structures exhibiting only slight filament merging alongside strong layer stacking and good shape fidelity. Furthermore, the cylindrical structures printed with “B2” showed good vertical fidelity, although the smoothness of structural edges could still be improved. These promising results suggest that the balance of 4% w/w gelatin and 2% w/w agarose provided sufficient viscosity for structural stability while maintaining extrudability, aided by the constant HPC content (2% w/w).^32^ Slight overextrusion was noted, but this can be addressed by adjusting parameters such as print speed to enhance cooling between layers.^33^ The pore area mismatch for “B2” was higher (7.05%) than the other samples, again due to thicker filament deposition, but this trade-off was accompanied by excellent reproducibility and layer stacking. Conversely, the other samples exhibited much higher RSD values and greater degrees of variability. Sample “A2” exhibited irregular filament diameters (RSD 1.08–7.15%), indicating partial printing consistency; however, the observed variability in filament deformation and merging, particularly in cylindrical constructs where layer collapse occurred, suggests a softer and less rigid material attributable to its higher gelatin content.^34^ Interestingly, while “A2” and “B2” prints appeared more visually uniform, their areas within the printed grid shapes were both smaller than the intended CAD target, not due to poor fidelity but rather because of thicker filaments reducing pore openings. This contrasted with “C2”, which produced thinner filaments and thus a pore area closest to the CAD target (1.82% mismatch). The highest variability was recorded for formulation “C1”, with RSD values for filament diameters between 15.9–16.7%. The irregular deposition and merging at intersections reflected poor filament control, which was likely due to the increased viscosity reducing responsiveness to pressure fluctuations during extrusion.^35^ Despite this poor reproducibility, the pore area mismatch for “C1” was relatively low (2.54%), which was attributable to thinner filament deposition rather than structural uniformity. These irregularities rendered “C1” the least reproducible of the test formulations, while formulation “C2” showed mixed performance, displaying excellent initial print uniformity with RSD values as low as 0.95% but increasing variability during longer print runs (up to 8.23%) due to the higher agarose content and associated viscosity, which caused brief nozzle clogging that reduced print resolution and compromised the cylindrical structures. Nonetheless, “C2” achieved the closest pore area match to the CAD design (1.82%), highlighting its potential for applications requiring accurate pore geometry, if process parameters are further optimized. Comparisons to the literature reinforce these findings. Wang et al. applied an iterative feedback printing strategy to gelatin/alginate bioinks, progressively reducing pore size mismatch from ∼ 40% in early prints to within ∼ 7% of the CAD target after optimization cycles.^36^ In contrast, the present work achieved pore area deviations as low as 1.82% for formulation “C2” and RSD values of 3.5–7.05% for the remaining formulations, all without iterative feedback, demonstrating the inherent reproducibility of the developed inks even prior to further optimization. Notably, these inks also exhibited electrical conductivity, extending their potential use to bioelectronic devices, biosensing platforms, and customized organ-on-chip systems requiring integrated electroactive functionality.^37^

Having established the rheological performance and print fidelity of the formulations, the next step was to evaluate their electrical properties, as the ability of a bioink to support charge transport directly influences its suitability for applications in signal transduction, electrochemical sensing, and bioelectronic integration.^38^ To this end, four-point probe analysis was employed to determine sheet resistance, resistivity, and conductivity, summarized in Table 3. All three blank samples exhibited high sheet resistance values, consistent with their inherently insulating nature. Among these, the “A” blank (5% w/w gelatin, 1% w/w agarose) recorded the highest sheet resistance (233.00 kΩ/sq) and resistivity (494.01 Ω·m), reflecting the dominance of the protein-rich matrix. Both the “B” and “C” blanks displayed lower values (44.40 and 54.40 kΩ/sq, respectively), likely due to their higher agarose content providing a limited degree of ionic conductivity.^39^ The incorporation of PEDOT:PSS markedly improved the conductive performance of the formulations. Sample “A2” (0.1% w/w PEDOT:PSS) exhibited the poorest results out of the samples containing PEDOT:PSS, with a sheet resistance of 7.93 kΩ/sq, a resistivity of 15.90 Ω·m, and a conductivity of 0.063 S/m. Conversely, “B2”, which contained the same concentration of PEDOT:PSS but was formulated with 4% gelatin and 2% agarose, demonstrated the most favorable conductive properties. It displayed the lowest sheet resistance (0.68 kΩ/sq), the lowest resistivity (1.74 Ω·m), and the highest conductivity (0.576 S/m) of all tested formulations. This suggested that the balanced polymer composition in “B2” supported a more effective dispersion of PEDOT:PSS and facilitated enhanced charge transport. Formulations “C1” and “C2” (3% gelatin, 3% agarose) further highlighted the role of matrix composition. “C1” showed moderate conductivity, while “C2” improved with higher PEDOT:PSS loading. These findings indicated that while excessive gelatin restricted chain mobility and reduced conductive pathways, increasing PEDOT:PSS loading partially overcame this limitation by creating more electronically conducting networks.^40^ Furthermore, the achieved conductivity of ∼0.576 S/m of sample “B2” aligns closely with biologically relevant conductive hydrogels, such as silk/PEDOT:PSS composites, which typically range between 0.2 and 1.2 S/m, which have been successfully applied to neural cell cultures and network formation.^41^ In contrast, higher values have been reported for specialized PEDOT:PSS hydrogels, including the ultrasoft adhesive platform for bioelectronics (4.43 S m^–1^) developed by Zhang et al.^42^ However, while these materials demonstrated excellent mechanical compliance and strong adhesion to porcine tissues, their assessment of biocompatibility was limited to indirect measures such as modulus matching and adhesion tests. No direct cell encapsulation, cytotoxicity, or viability assays were performed, in contrast to the present work, where biocompatibility was confirmed through MTS and LDH assays across multiple cell–hydrogel interaction conditions. The current formulations provide a more comprehensive bioink platform by combining conductivity, print fidelity, and verified cytocompatibility, a difficult balance to achieve in electrically conductive hydrogels. Although the conductivities are lower than those required for high-current electrochemical devices, they fall within biologically relevant ranges and, crucially, are achieved alongside robust biocompatibility and reliable printability, both of which have been rigorously validated in this work. This combination of tunable conductivity, suitable viscosity, and demonstrated cell compatibility positions the materials as versatile candidates for a wide range of biofabrication applications.

The ultimate compressive strength, elastic modulus, and toughness were determined for “B2” as summarized in Figure 4. Three different conditions were considered, focusing on conditions relevant to its intended applications: freshly prepared at room temperature (dry), wetted, and submerged for a time. Hydration significantly increased the elastic modulus and compressive strength compared to the dry state (*p* < 0.05). However, there was no significant difference (*p* = 0.32) between the wetted and submerged conditions, indicating that most of the stiffening effect occurs upon initial hydration. This behavior mirrors observations in other protein–polysaccharide hydrogels, where swelling facilitates chain mobility and improved load distribution.^43,44^ The increase in toughness, most evident under submerged conditions, highlights the role of gelatin in dissipating energy through reversible hydrogen bonding, consistent with previous reports of gelatin-based hydrogels.^45^ A statistically significant difference was observed only between the dry and submerged states (*p* = 0.007), indicating that full hydration is required to achieve a substantial improvement in energy absorption. Meanwhile, the hydrophilic nature of HPC likely facilitated water ingress and stress relaxation, a mechanism also described in cellulose-derived hydrogels.^46^ The results observed between the mechanical characterization and conductivity of “B2” position it as a strong candidate for next-generation bioelectronic platforms, where soft, tissue-like scaffolds are essential for seamless integration in biosensing and implantable devices.^47^

Figure 5 shows SEM images for the “B2” bioink formulation at different magnifications. While recognizing that EtOH dehydration can alter the structure of hydrogels,^48^ the dehydrated material demonstrated a highly interconnected porous network. Such porosity is advantageous for both fluid and analyte transport, as well as for supporting cell growth, features widely recognized as critical in 3D biosensing and tissue engineering scaffolds.^49^ The observed pore dimensions (40–100 μm) are comparable to those reported for other protein–polysaccharide hydrogels and fall within ranges that allow effective analyte diffusion and potential cell infiltration. Trifonov et al. reported that hydrogels used in tissue engineering typically exhibit interconnected pore diameters spanning 1–250 μm, a range that encompasses the dimensions measured here and reinforces their suitability for supporting mass transport and cellular integration.^50^ At higher magnifications (50 μm and 40 μm; Figure 5A and 5B), the surface displayed a heterogeneous and rough morphology. Such microscale roughness is commonly associated with phase separation or variations in cross-linking density during gel formation, features that are often accentuated by ethanol dehydration.^51^ At lower magnification (100 μm; Figure 5C), the interconnected pore network was clearly visible, while the cross-sectional view (400 μm; Figure 5D) highlighted the internal architecture supporting a uniform distribution of hydrogel components. In addition, the micro- to mesoscale porosity observed in the printed structures further supports their suitability for 3D tissue scaffold applications in regenerative medicine, where interconnected pores are essential for promoting cell infiltration, nutrient exchange, and overall tissue integration within electrically conductive environments.^52^

Electrochemical Impedance Spectroscopy (EIS) was used to probe the dynamics of heterogeneous electron transfer across the printed bioink/solution interface. Here, the “B2” bioink was printed onto a screen-printed carbon electrode (SPCE) that acted as a conducting support. Ferricyanide (Fe­(CN)_6_^3–^) was used as the negatively charged redox active probe, which could electrostatically associate with the slightly positively charged “B2” structure, thereby enhancing sensitivity to changes in the rate of interfacial electron transfer. Figure 6 indicates that the unmodified SPCE displayed the largest semicircle, corresponding to the highest charge transfer resistance (*R*_ct_ ∼ 1.2 kΩ) and the slowest electron exchange at the interface, while the base hydrogel without PEDOT:PSS exhibited the smallest semicircle (*R*_ct_ ∼ 220 Ω), reflecting facilitated ionic transport through the hydrated polymer matrix. Interestingly, incorporation of 0.1% PEDOT:PSS in the “B2” increased *R*_ct_ (∼522 Ω) relative to the “B” base. This effect is likely a result of the denser cross-linked structure formed by ionic cross-linking between gelatin’s guanidino groups and PSS’s sulfonate moieties.^13^ Furthermore, “B2” exhibited the greatest degree of swelling among the “B” formulations, suggesting that increased water uptake leads to a thicker hydrated layer on the electrode surface and contributes to the observed increase in impedance.^53^ This denser network reduces the pore size and restricts the mobility of ionic species within the hydrogel, demonstrated by the longer and more pronounced Warburg line indicating greater diffusion impedance.^54^ In addition, the negative charge of PSS likely introduced electrostatic repulsion with the Fe­(CN)_6_^3–^/^4–^ couple, further slowing electron transfer and contributing to the observed rise in the interfacial resistance. Comparable approaches have been reported by Furlani et al., who used EIS to evaluate gelatin-PEDOT:PSS hydrogels cross-linked with genipin for nervous tissue regeneration.^13^ Their analysis focused on bulk conductivities in the range of 10^–3^–10^–2^ S m^–1^, derived from Nyquist plots, confirming the contribution of PEDOT:PSS to charge transport within hydrated gelatin matrices. In the present work, EIS provided complementary insight into interfacial charge transfer, while four-point probe measurements demonstrated that incorporation of PEDOT:PSS substantially enhanced the overall electronic conductivity, reaching ∼0.576 S m^–1^ for “B2”. Together, these results established that PEDOT:PSS improved charge transport at both the bulk and interfacial levels, strengthening the case for their applications in electrically integrated biofabrication.^55^

For testing the biocompatibility of “B2”, one-way ANOVA was applied to examine the effect of time on cell viability within each material condition. Across all conditions, “B2” was the only material with *p* < 0.05, indicating a potential time effect; however, posthoc pairwise comparisons revealed no significant differences between individual time points, suggesting that the observed variability was distributed rather than driven by a distinct time-dependent drop. For all other materials, including the base hydrogel and the untreated control, no significant changes were observed over 72 h (*p* > 0.05), supporting stable compatibility across the assay period. A separate one-way ANOVA was then performed to compare between material types at each individual time point. As expected, DOX-containing samples differed notably from their non-DOX counterparts, reflecting the known cytotoxic effect of doxorubicin. By contrast, posthoc analysis confirmed non-DOX comparisons (e.g., Control vs “B”, Control vs “B2”, “B” vs “B2”) were not significant, indicating similar biocompatibility between untreated formulations. These findings highlight two key points: (i) untreated hydrogels maintain high viability over 72 h with no statistically meaningful decline (*p* > 0.05) and (ii) DOX loading uniformly reduces viability across all formulations in a manner consistent with its therapeutic cytotoxicity, without additional detrimental effects attributable to PEDOT:PSS or hydrogel composition at the concentrations used. This behavior is in line with reports that gelatin/agarose bioinks preserve short-term viability and that low PEDOT:PSS loadings (≤0.1% w/w) are compatible with mammalian cells when embedded in hydrated polymer networks.^56^ The LDH assay supported these trends. One-way ANOVA detected *p* < 0.05 only for “B2”, but posthoc tests again found no significant pairwise differences between time points, indicating minor, evenly distributed variability. Between-material comparisons confirmed significantly higher cytotoxicity in all DOX groups at each time point, while non-DOX groups were indistinguishable. Therefore, these results demonstrate that the base and PEDOT:PSS-containing hydrogels were noncytotoxic over 72 h, and DOX effects were material-independent. Calcein-AM images (Figure 8) provided a visual correlate to the quantitative assays. Robust green fluorescence was observed for Control, “B”, and “B2” across all configurations (“Under,” “Inside,” “In-between,” “On”), and no visible differences were noted between Control and “B/B2”, indicating that the bioink did not depress cell numbers under the test conditions. In contrast, DOX-treated samples displayed markedly reduced fluorescence in every configuration, consistent with the expected cytotoxic profile. These qualitative observations were further supported by fold-change analysis of the calcein fluorescence area, which demonstrated comparable values for Control and bioink conditions and a pronounced reduction in all DOX-containing groups (Table S2). Similar systems such as the agarose-based bioink developed by Gu et al. reported an initial high cell viability but then a reduction in cell survival over the first few days, with values declining to ∼71% by Day 4 before stabilizing at ∼74% on Day 7.^31^ By contrast, in the present study, untreated hydrogel formulations (Control, “B”, “B2”) consistently maintained high cell viabilities above 90% at 72 h across all experimental conditions (Figure 7i–iv), with no significant time-dependent cell toxicity detected. Importantly, these outcomes were achieved in a thermoresponsive system that also incorporated PEDOT:PSS, introducing electrical conductivity and thereby extending the functionality of the hydrogel beyond biocompatibility alone. The ability to combine printability, biocompatibility, and conductivity within a single hydrogel system is particularly significant, as it opens the pathway for applications from biosensing to electroactive scaffolds.^57^

## 5 Conclusion

This study reports a novel, multicomponent electrically conductive, thermoresponsive family of bioinks, with particular focus on formulation “B2” (4% w/w gelatin, 2% w/w agarose and 2% w/w HPC, 0.1% w/w PEDOT:PSS). The purpose of this work was to develop and critically evaluate a bioink system that balances mechanical performance, rheological behavior, electrical conductivity, printability, and biocompatibility using an integrated approach that is rarely undertaken in conductive hydrogel research, where studies often prioritize only one parameter at a time (e.g., conductivity, print fidelity, or cell compatibility). By characterizing all these properties systematically, this work establishes a multifunctional bioink platform with broad translational potential. Swelling and degradation studies indicated that “B2” exhibited enhanced water retention and mechanical stability. Electrical analysis reinforced the multifunctionality of “B2”. Four-point probe measurements demonstrated a conductivity of ∼0.576 S/m, placing it within the range of biologically relevant conductive hydrogels and outperforming comparable gelatin–PEDOT:PSS systems reported in the literature. Electrochemical impedance spectroscopy provided complementary insights, showing increased interfacial resistance due to the denser network and electrostatic repulsion from PSS sulfonates, yet corroborating that PEDOT:PSS incorporation established electronic conduction pathways throughout the bulk hydrogel. Rheological characterization confirmed that “B2” achieved the most favorable balance between elasticity and recovery after shear, while SEM, compression tests, and printability testing confirm that the printed structures have both micro- and mesoporosity and excellent mechanical stability and tunability. Importantly, both the MTS and LDH assays demonstrated that the presence of the hydrogel, with or without PEDOT:PSS, did not impact cell viability or cytotoxicity after 72 h, confirming the intrinsic biocompatibility of the formulations. These findings are further supported by the fluorescence microscopy images, which qualitatively showed live cells distributed throughout the hydrogel matrices under all experimental configurations. A limitation of the present study is that biocompatibility testing was restricted to a single cell line and a short-term culture. However, given the intrinsically biocompatible nature of the gelatin-agarose-HPC matrix, there is strong potential to extend these findings to a broader range of primary and specialized cell types. Future work will focus on longer-term viability and proliferation studies, the use of wider cell panels to evaluate tissue-specific compatibility, and the incorporation of sensing elements such as antibodies to develop functional biosensors. Furthermore, while “B2” was identified as the optimal formulation for this study, the other inks developed here, such as the stiffer agarose-rich or more gelatin-rich variants, could be further optimized for alternative applications where their mechanical or swelling properties may offer advantages, such as load-bearing scaffolds or drug delivery platforms. To conclude, this study establishes a comprehensive and systematic framework for formulating thermoresponsive electrically conductive bioinks, integrating swelling and degradation analysis, rheological profiling, printability testing, and detailed mechanical, structural, electrical, and biocompatibility characterization. Achieving simultaneous thermoresponsiveness, electrical conductivity, printability, and biocompatibility is rarely accomplished in bioink development, yet the “B2” formulation demonstrated an exceptional balance of these competing properties. As one of the few studies to successfully formulate this combination, these findings offer a uniquely versatile material platform that can be further utilized for advanced tissue engineering, electroactive cell culture systems, and emerging biosensing or bioelectronic applications, as well as future integration into more complex functional constructs.

## Supplementary Material



## Notes

Swelling analysis data for all bioink formulations (Table S1); quantitative fold-change analysis of fluorescence intensity (Table S2); schematic illustration of experimental setups for cell viability testing (Figure S1); amplitude sweep rheological data for blank and bioink samples (Figure S2); viscosity as a function of temperature for all formulations measured in triplicate (Figure S3) (PDF)
