# Supplementary material for: A Thermoresponsive, Electrically Conductive Bioink Optimized for Electroactive Tissue Engineering and Bioelectronics
Source: ACS Appl Bio Mater. 2026 Feb 15;9(6):2895–913. doi: 10.1021/acsabm.5c02097 (PMC12997253; doi:10.1021/acsabm.5c02097)
Supplement: Supplementary file 1 [file mt5c02097_si_001.pdf]

Supporting Information

A Thermoresponsive, Electrically Conductive Bioink Optimised for Electroactive Tissue Engineering and Bioelectronics

Roisin Byrne [1], John Redmond [2], Keith D. Rochfort [3,4**], Amanda Carrico[1,5], Robert J Forster [1,5], Nicholas Dunne [2,6-10], and Loanda R Cumba [1*]

[1] School of Chemical Sciences, Dublin City University, Glasnevin, Dublin 9, D09 E432, Ireland

[2] Centre for Medical Engineering Research, School of Mechanical and Manufacturing Engineering, Dublin City University, D09 NA55, Dublin, Ireland

[3] School of Biotechnology, Dublin City University, Glasnevin, Dublin 9, Ireland

[4] Life Sciences Institute, Dublin City University, Dublin 9, Ireland

[5] FutureNeuro, The SFI Research Centre for Chronic and Rare Neurological Diseases, Royal College of Surgeons in Ireland.

[6] Biodesign Europe, Dublin City University, Dublin 9, Ireland

[7] School of Pharmacy, Queen's University of Belfast, 97 Lisburn Road, Belfast BT9 7BL, UK

[8] Advanced Manufacturing Research Ireland Centre (I-Form), School of Mechanical and Manufacturing Engineering, Dublin City University, Dublin 9, Ireland

[9] Advanced Materials and Bioengineering Research Ireland Centre (AMBER), Royal College of Surgeons in Ireland and Trinity College Dublin, Dublin 2, Ireland

[10] CÚRAM, Research Ireland Centre for Medical Devices, University of Galway, Galway, Ireland

*Corresponding Author: loanda.cumba@dcu.ie

**Corresponding Author: keith.rochfort@dcu.ie

Table S1. Swelling analysis results. Values represent the percentage increase in weight relative to the initial dry weight at 0 h

Sample	Degree of Swelling (%)								
	**1h**	**2h**	**3h**	**4h**	**5h**	**15h**	**24h**	**48h**	**72h**
**A1**	137.3	217.3	242.4	267.2	277.9	394.5	413.4	415.4	428.1
**A2**	493.1	565.6	640.0	665.2	694.4	725.9	796.0	804.6	809.6
**A3**	392.6	463.9	495.9	512.2	562.7	678.0	684.7	683.6	684.8
**B1**	108.4	154.5	174.8	191.5	197.2	220.0	384.8	378.3	380.1
**B2**	118.0	149.5	168.6	186.9	194.1	342.0	400.5	403.1	405.6
**B3**	72.7	265.4	284.1	292.2	298.3	411.3	416.6	402.3	399.0
**C1**	146.4	256.6	265.0	284.2	292.9	522.9	561.2	570.6	576.9
**C2**	213.9	263.0	269.6	299.8	308.5	388.8	379.6	385.7	380.8
**C3**	406.8	389.4	451.7	451.8	437.5	442.3	440.9	434.4	435.2

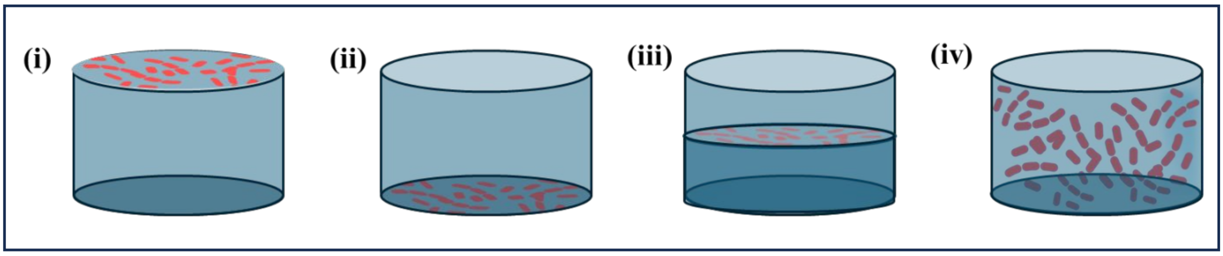

Figure S1: Schematic representation of the four experimental setups for cell viability testing. (i) ‘On’: Cells were placed on top of the hydrogel sample. (ii) ‘Under’: Cells were deposited beneath the hydrogel sample. (iii)’ Inbetween’: Cells were deposited on one half of the hydrogel and pressed with the other half (to ensure proper integration, the top hydrogel layer was added in a molten state and allowed to set, fusing both layers together). (iv) ‘Inside’: Cells were mixed uniformly within the hydrogel matrix.

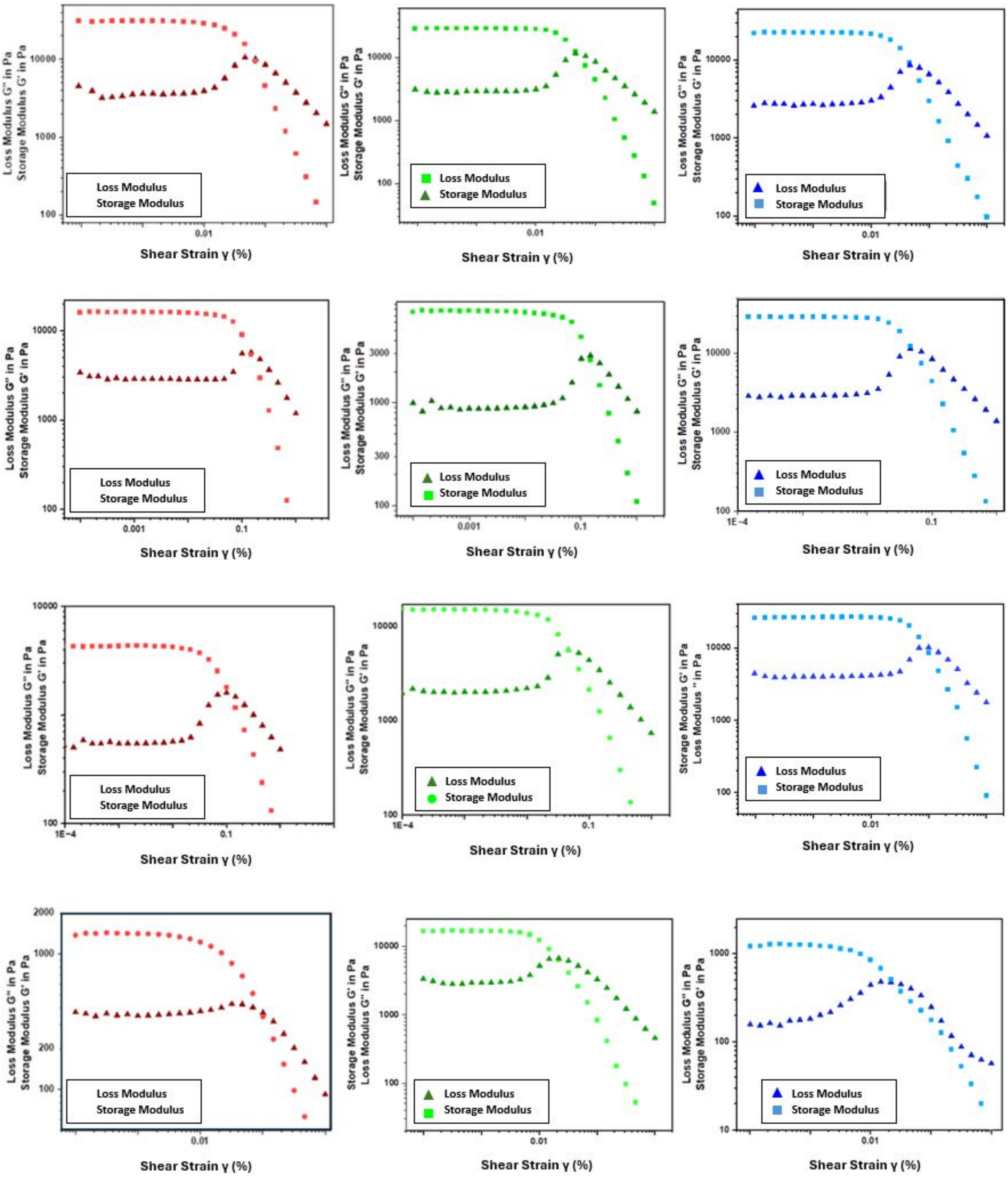

Figure S2. Amplitude sweeps of row one blank sample 'A', blank 'B' and blank 'C'. Row two denotes sample 'A1', 'B1', 'C1', row three denotes sample 'A2', 'B2', 'C2', and row four shows samples 'A3', 'B3', and 'C3' respectively.

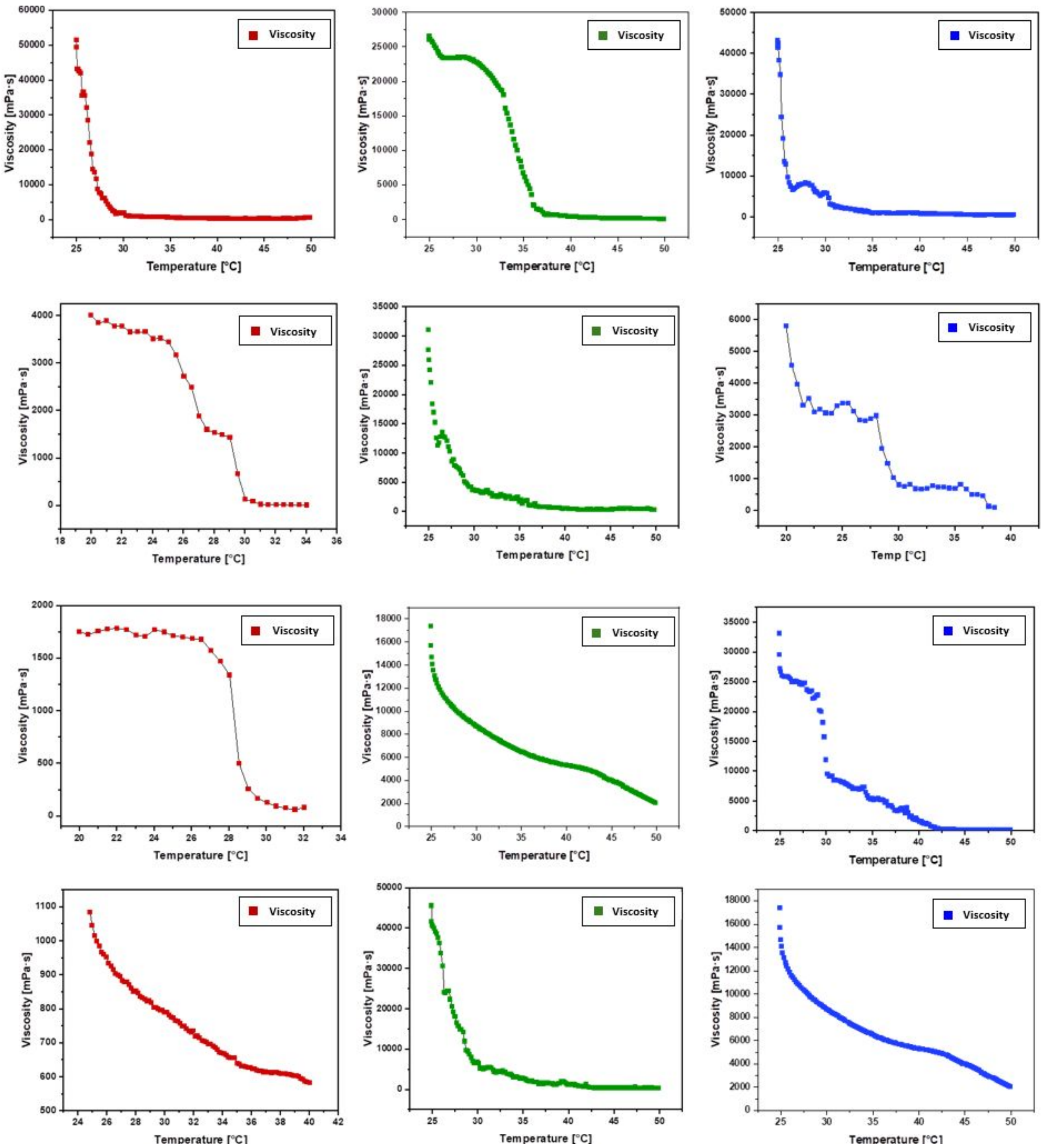

Figure S3. Viscosity vs. Temperature of row 1 blank sample ‘A’, blank ‘B’ and blank ‘C’. Row 2 denotes sample ‘A1’, ‘B1’, ‘C1’, row 3 denotes sample ‘A2’, ‘B2’, ‘C2’, and row 4 shows samples ‘A3’, ‘B3’, and ‘C3’ respectively. Measurements were performed in triplicate (n = 3); plotted data represent mean values.

Table S2. Fold change (mean ± SD) in calcein fluorescence intensity under different hydrogel spatial configurations and treatment conditions.

Conditions	Control	B	B2	Control/Dox	B/DOX	B2/DOX
**'Under'**	1.00 ± 0.09	1.02 ± 0.07	1.05 ± 0.08	0.27 ± 0.03	0.29 ± 0.01	0.29 ± 0.06
**'Inside'**	1.00 ± 0.11	1.07 ± 0.18	1.05 ± 0.15	0.25 ± 0.05	0.27 ± 0.04	0.27 ± 0.03
**'Inbetween'**	1.00 ± 0.09	1.05 ± 0.09	1.02 ± 0.07	0.30 ± 0.03	0.31 ± 0.03	0.299 ± 0.05
**'On'**	1.00 ± 0.09	1.05 ± 0.17	1.06 ± 0.05	0.26 ± 0.02	0.29 ± 0.01	0.28 ± 0.05
